# Pharmacist-Led Medication Reviews to Identify and Collaboratively Resolve Drug-Related Problems in Psychiatry – A Controlled, Clinical Trial

**DOI:** 10.1371/journal.pone.0142011

**Published:** 2015-11-06

**Authors:** Carolin Wolf, Anne Pauly, Andreas Mayr, Teja Grömer, Bernd Lenz, Johannes Kornhuber, Kristina Friedland

**Affiliations:** 1 Molecular & Clinical Pharmacy, Friedrich-Alexander University Erlangen-Nürnberg (FAU), Erlangen, Germany; 2 Department of Medical Informatics, Biometry and Epidemiology, Friedrich-Alexander-University Erlangen-Nürnberg (FAU), Erlangen, Germany; 3 Department of Psychiatry and Psychotherapy, Friedrich-Alexander University Erlangen-Nürnberg (FAU), Erlangen, Germany; University of Melbourne, AUSTRALIA

## Abstract

**Aim of the study:**

This prospective, controlled trial aimed to assess the effect of pharmacist-led medication reviews on the medication safety of psychiatric inpatients by the resolution of Drug-Related Problems (DRP). Both the therapy appropriateness measured with the Medication Appropriateness Index (MAI) and the number of unsolved DRP per patient were chosen as primary outcome measures.

**Methods:**

Depending on their time of admission, 269 psychiatric patients that were admitted to a psychiatric university hospital were allocated in control (09/2012-03/2013) or intervention group (05/2013-12/2013). In both groups, DRP were identified by comprehensive medication reviews by clinical pharmacists at admission, during the hospital stay, and at discharge. In the intervention group, recommendations for identified DRP were compiled by the pharmacists and discussed with the therapeutic team. In the control group, recommendations were not provided except for serious or life threatening DRP. As a primary outcome measure, the changes in therapy appropriateness from admission to discharge as well as from admission to three months after discharge (follow-up) assessed with the MAI were compared between both groups. The second primary outcome was the number of unsolved DRP per patient after completing the study protocol. The DRP type, the relevance and the potential of drugs to cause DRP were also evaluated.

**Results:**

The intervention led to a reduced MAI score by 1.4 points per patient (95% confidence interval [CI]: 0.8–2.0) at discharge and 1.3 points (95% CI: 0.7–1.9) at follow-up compared with controls. The number of unsolved DRP in the intervention group was 1.8 (95% CI: 1.5–2.1) less than in control.

**Conclusion:**

The pharmaceutical medication reviews with interdisciplinary discussion of identified DRP appears to be a worthy strategy to improve medication safety in psychiatry as reflected by less unsolved DRP per patient and an enhanced appropriateness of therapy. The promising results of this trial likely warrant further research that evaluates direct clinical outcomes and health-related costs.

**Trial Registration:**

Deutsches Register Klinischer Studien (DRKS), DRKS00006358

## Introduction

As a result of the high prevalence of risk factors, such as polypharmacy commonly applied by multiple prescribers, several comorbidities and inadequate adherence, psychiatric patients are at significant risk for Drug-Related Problems (DRP) [1]. DRP comprise both non-preventable Adverse Drug Events (ADE) and errors in medication therapy that differ in their actual or potential risks to cause patient harm [2]. DRP are directly associated with impaired health outcomes, such as the worsening of symptoms or a prolonged hospital stay [3]. There is evidence for an increased prevalence of ADE and medication errors in the psychiatric setting compared with other medical conditions [4–6], which substantially endanger the medication safety of psychiatric patients [7]. An increased awareness regarding the safety of drug therapy emerged in the psychiatric setting with a Task Force on Patient Safety published by the American Psychiatric Association (APA) [7]. The implementation of strategies to identify, report and prevent medication errors was recommended as one of the most important activities to improve the safety of psychiatric patients.

Despite the recommendations by the APA, only limited research is available regarding prevention strategies of ADE and medication errors in psychiatric patients [1,5,6,8–12]. An interdisciplinary attempt that implemented structured pharmaceutical medication reviews with subsequent changes in therapy to optimize pharmacological treatment in the hospital has been demonstrated to improve medication safety in trials predominantly conducted in internal or geriatric medicine [13,14]. The error ratios were reduced during the hospital stay, and the clinical outcomes were improved. Although there is also some evidence that indicates the effectiveness of pharmacy team led medication reviews and medication reconciliation to detect and solve DRP in psychiatry, the available trials are small and lacked a control group [15–19].

Therefore, we aimed to assess the feasibility and the impact of structured, comprehensive medication reviews by clinical pharmacists including the subsequent interdisciplinary discussion of identified DRP on the drug safety of psychiatric inpatients compared with usual psychiatric care in a German university hospital in a prospective, controlled trial. The appropriateness of the therapy, which was measured with the Medication Appropriateness Index (MAI) [20] at admission, discharge and three months after discharge, and the number of unsolved DRP were chosen as the primary outcomes because all efforts to enhance the safety of drug therapy address these parameters [21]. DRP were branded as unsolved if the physicians did not implement the pharmaceutical recommendations or if a pharmaceutical intervention was accepted, but had not been implemented after the entire study period. Furthermore, the study intended to assess the DRP type, outcome and relevance, as well as the potential of commonly prescribed drugs to cause DRP. We demonstrated the opportunity of pharmaceutical medication reviews with subsequent interdisciplinary discussion of identified DRP to enhance medication safety in psychiatry.

## Methods

### Setting and participants

This prospective, non-randomized, open, controlled study was conducted on two non-acute wards in the Department of Psychiatry and Psychotherapy at the University Hospital of Erlangen. Both wards were used as control and intervention wards consecutively. The medication orders were paper-based. Only centralized pharmaceutical services were available by telephone. Pharmaceutical medication reviews were not performed regularly on the study wards prior to the start of the control phase. The detailed pharmaceutical reviews on admission, discharge and post-discharge were firstly implemented in the control phase and were extended during the intervention phase of this study. Usual care for patients admitted to the Department of Psychiatry and Psychotherapy contained consultation of a physician and of a senior psychiatrist once weekly as well as a group counseling once weekly on alternating topics regarding the psychiatric diseases (e.g. symptoms of disease, coping with disease).

All patients aged 18 and older who were admitted to one of the two wards between September 2012 and March 2013 (control phase) or May and December 2013 (intervention phase) were eligible for inclusion in the control group or the intervention group, respectively. A randomized, parallel group design was not applied because of the knowledge bias generated in the control group when pharmaceutical recommendations are implemented in the patients in the intervention group on the same ward. Therefore, a consecutive design was conceived. The mean length of stay in the Department of Psychiatry and Psychotherapy was stated to be 21 days. Accordingly, a wash-out period of one month between the two phases should have ensured that all patients in the control group were discharged prior to the start of the intervention. Additional inclusion criteria included the ability to understand and write in German, a psychiatric hospital stay of more than six days and the consent to be contacted after discharge. Patients were excluded if they did not take psychiatric medication. A few days after admission, the patients were fully informed about the content and objectives of the study. Afterwards, written informed consent was obtained for those patients willing to take part in the trial.

### Ethics Statement

The procedures were in concordance with the ethical standards of the Ethics Committee of the Friedrich-Alexander University Erlangen-Nürnberg, which also approved the study protocol at 26 July 2012 (174_12B). No significant changes were made to the study protocol after approval of the ethical committee. The trial was registered with the German register of clinical studies (DRKS00006358, https://drks-neu.uniklinik-freiburg.de/drks_web/navigate.do?navigationId=trial.HTML&TRIAL_ID=DRKS00006358). The study was registered after enrolment of patients started because the responsible persons were not aware of the need to register before. The full trial protocols in German and English are attached as supplemental files (S1 Protocol and S2 Protocol). The authors confirm that all ongoing and related trials for this intervention are registered.

### Definitions

According to the Pharmaceutical Care Network (PCNE), a Drug-Related Problem is “an event or circumstance involving drug therapy that actually or potentially interferes with desired health outcomes” [22]. DRP comprise medication errors and ADE [23]. ADE are described as “any injury resulting from medical interventions related to a drug” [24]. A medication error is “any preventable event that may cause or lead to inappropriate medication use or patient harm while the medication is in the control of the health care professional, patient, or consumer” [25]. Medication errors differ in their potential or actual risks for patient harm [2]. They are categorized as having little or no risk for patient harm or a potential risk while the patient has not yet been injured (potential ADE). Additionally, errors in the medication process that harmed patients are referred to as preventable ADE [2]. ADE that were not associated with medication errors are regarded as non-preventable [2].

### Intervention

After eligible patients gave written consent to take part in the study, both patients in the control group and the intervention group obtained detailed medication reconciliation at admission and medication reviews at discharge and three months after discharge, as well as weekly during the hospital stay by two clinical pharmacists. The reviews at admission, discharge and follow-up included a comprehensive patient interview and the assessment of drug history and ADE by two pharmacists. A standardized form was utilized to assess trade names, dosage, indication, application, duration of use and tolerability of the applied drugs, as well as the patient characteristics, including diagnoses, allergies and laboratory parameters. The medication reviews were conducted subsequent to the physicians’ interviews shortly after admission and shortly before discharge. The noted medication charts were checked for interactions with two drug-drug interaction programs [26,27]. DRP were assessed according to a structured checklist, which included the items indication, effectiveness, dosage, directions, practicability, drug-drug interactions, contraindications, duplication, duration of therapy, side effects, compliance, untreated indication and monitoring. The weekly hospital medication reviews were performed under consideration of laboratory parameters. Furthermore, the pharmacists participated in multidisciplinary ward rounds (six times per week).

Recommendations for all identified DRP were compiled immediately after their identification regardless of their severity for patients in the intervention group. The appropriate advices were orally disseminated to the ward staff, discussed with the attending physicians and nurses and implemented if possible within the same day of identification. Recommendations regarding DRP that addressed practical and correct directions or knowledge regarding the individual therapy were also discussed with the patients. Changes in therapy were communicated to the patients by the pharmacists or ward staff. Recommendations for identified DRP of patients in the control group were only disseminated to medical staff if they were serious or life threatening because of ethical considerations.

Furthermore, the patients of the intervention group additionally obtained pharmaceutical counseling during the hospital stay with individual drug information leaflets regarding their psychiatric disease and drugs. A discharge medication plan was given and explained to the patients. The patients in the intervention group were also contacted by telephone after discharge at 1.5 and 6 weeks to discuss and solve ongoing problems with their medication.

Besides the comprehensive medication reviews at admission, discharge and follow-up patients in the control group received usual psychiatric care as described above.

The pharmacists that were responsible for identifying DRP and compiling recommendations were licensed, but did not work in a psychiatric hospital before. Prior to the start of the study, the two pharmacists were first trained intensively regarding psychiatric diseases and medication. Afterwards they joined for three months the physiatrists that were working on the wards.

### Outcome measure

The primary outcome measures were the change of therapy appropriateness measured by the MAI [20] between admission and discharge and admission and follow-up, as well as the number of unsolved DRP per patient after the entire study period. The number of DRP per 1,000 patient-days was assessed for comparison with other medication safety studies in psychiatry, which have commonly used this denominator [5,8,9]. The relevance of the identified DRP, the potential of commonly applied drugs to cause DRP and the categorization of DRP and their outcomes according to the Problem-Intervention-Outcome (PIO) classification system were evaluated as the secondary outcomes.

### The MAI

The MAI includes ten implicit and explicit criteria to review the appropriateness of each prescribed drug regarding the indication, effectiveness, dosage, correct directions, practical directions, drug-drug interaction, drug-disease interaction, duplication, duration and expense. A weighted scoring system was applied in accordance with Samsa et al [28]. Each question per drug is rated as appropriate, marginally appropriate or inappropriate. In this trial, the rating was dichotomized to appropriate (appropriate and marginally appropriate), which indicates zero points in the scoring system, and inappropriate. A criterion that is rated inappropriate receives points according to its importance for the appropriateness of therapy, and more points indicate an increased importance. For example, a drug without a clear indication was rated three points. In contrast, an inappropriate duration of drug therapy was assigned one point. Therefore, a maximum of 18 points per drug could be obtained, whereas a higher sum score per drug represents increased inappropriateness. The assessment of appropriateness with the MAI requires detailed clinical information. Nevertheless, when applied in the hospital setting, the inter- and intra-rater reliabilities were evaluated as good [20,29].

### Data Collection

The demographic data included gender, age, number of prior psychiatric hospital stays, wards, length of hospital stay and number of drugs at admission, discharge and follow-up.

The MAI score was calculated on admission, discharge and follow-up. For the estimation of the weighted MAI scores at admission and discharge, the medical records documented in the patient charts at the time of the pharmaceutical medication review shortly after admission and shortly before discharge were used. To assess the follow-up value of the MAI score, the patients in both groups were contacted three months after discharge by telephone, and their medication plan was assessed by the two pharmacists. The patient MAI score was calculated via the summation of the weighted MAI scores for each noted drug the patient received at admission, discharge and follow-up, respectively. The subtraction of the patient MAI score at discharge and follow-up from admission resulted in the assessment of the change in patient MAI score. The MAI scoring was verified independently by a senior psychiatrist.

For the estimation of the number of unsolved DRP, all DRP that were identified during the hospital stay by the standardized pharmaceutical medication reviews in control group and intervention group were documented and branded as solved or unsolved depending on whether they were elucidated. Usually, if the physician agreed with the pharmacist’s recommendation, the changes were immediately transferred to patient charts. Occasionally, the medical records were not available or the psychiatrist firstly wanted to discuss the changes in therapy with the patient before they were implemented. In that case, the pharmacists afterwards checked the medical charts of respective patients to verify that the recommendations were implemented. DRP were also marked as solved if the recommended intervention was implemented after discharge but within the study period, which terminated three months after discharge. If a recommendation was not accepted for any reason or accepted but not certainly implemented, the DRP were labeled as unsolved. Furthermore, if a recommended intervention was accepted, but it had not been implemented within the entire study period, it was also marked as unsolved. Only DRP that were identified during the hospital stay were included because the pharmacists did not have access to valid medical information after patient discharge.

The identified DRP were classified according to the three-parted Problem-Intervention-Outcome (PIO) system [30], which is akin to the DRP classification of the Pharmaceutical Care Network Europe (PCNE) [22]. The PIO classification system developed in Mainz, Germany is included in a Microsoft access^®^-database (APOSTAT) [31]. The PIO system has been demonstrated to be reliable according to its internal and external reliabilities [30,32]. Based on the PI-Doc^®^ system [33], which has been adapted for a hospital setting, DRP can be categorized into 61 individual problem classifications. If the ward staff or pharmacists resolved a DRP, the observed outcome was classified as “improving patients’ safety”, “improving effectiveness of drug therapy”, “reducing medication costs”, “improving patient compliance/satisfaction” or “negative outcome”. The PIO system was selected because of its comprehensiveness and the lack of a valid result classification of the other classification systems in German [30]. Additional information regarding the DRP, including the causal drug, acceptance of recommendations and relevance, were also documented in the APOSTAT [31] database and were evaluated. The relevance of the DRP were estimated as minor, moderate or high in concordance with a previous pharmaceutical care project in psychiatry [34]. The potential of medications to cause DRP was calculated by dividing the number of DRP that were triggered by the respective drug by its number of prescriptions at admission.

The classification and relevance of the identified DRP were also verified independently by the senior psychiatric physician who was not working on the study wards. If the estimation of the extern reviewer deviated from the pharmacists’ evaluation, classification and relevance of DRP were discussed.

### Statistical analysis

Only the patient MAI score was used to perform the power calculation because no data were available for the number of unsolved DRP. According to Hanlon et al [35], an effect size of the intervention of 0.4 was assumed. To attain 80% power to detect an effect size of 0.4 with a two-tailed significance level of 0.05, 100 patients were required. To negotiate an attrition of 15% of patients because of drop-outs and 15% of patients because of loss to follow-up, a sample size of 130 patients per group was considered.

The patient characteristics, as well as the primary and secondary endpoints at the different time points were presented as the mean (± standard deviation, SD) or median (interquartile range, IQR) for the continuous variables and as numbers (%) for the categorical variables. As the group allocation was not random but assigned by the time of admission, the group differences at baseline were assessed via Chi squared or Fisher’s exact tests for the categorical variables and Student’s t-test or Mann-Whitney-U test for the continuous variables. Following the intention-to-treat principle, only the patients who were discharged prior to the first pharmaceutical interview were excluded from the analysis.

To investigate the efficacy of the intervention on the primary endpoints, we performed a regression analysis to adjust for group differences at admission. For the number of unsolved DRP, which represent the count data, a generalized linear model for the negative binomial distribution with log-link was computed. The number of unsolved DRP served as the response variable; the treatment group, sex, age, gender, comorbidities, number of drugs, length of hospital stays and total number of DRP were included as predictors. Thus, the group variable coefficient represents an adjusted treatment effect and was reported with the 95% confidence interval (CI) and corresponding p-value comparing the effect to 0.

For the patient MAI score, which was assessed at admission, discharge and follow-up, we applied a linear mixed-effect model approach to adjust for the longitudinal structure of the data. The outcome variable was the MAI score at the three different time points. This longitudinal modeling approach builds up a design matrix with one row for each measurement instead of for each patient; therefore, a missing outcome at follow-up (e.g., if the patient did not take medication at the third interview) does not lead to the deletion of the patient. As predictors, we included the same variables as for the DRP, with an additional interaction between the treatment group and time categories. Thus, the coefficient for the treatment x time interaction represents the adjusted effect of the intervention at the different time points and was reported with the 95% CI and corresponding p-value.

Statistical analyses were performed using IBM SPSS Statistics for Windows Version 22.0 (SPSS, IBM Corp., Armonk, New York, USA) and the statistical programming environment R 3.0.2 (R Foundation for Statistical Computing, Vienna, Austria, http://www.R-project.org/).

## Results

### Patient characteristics

**Fig 1:**
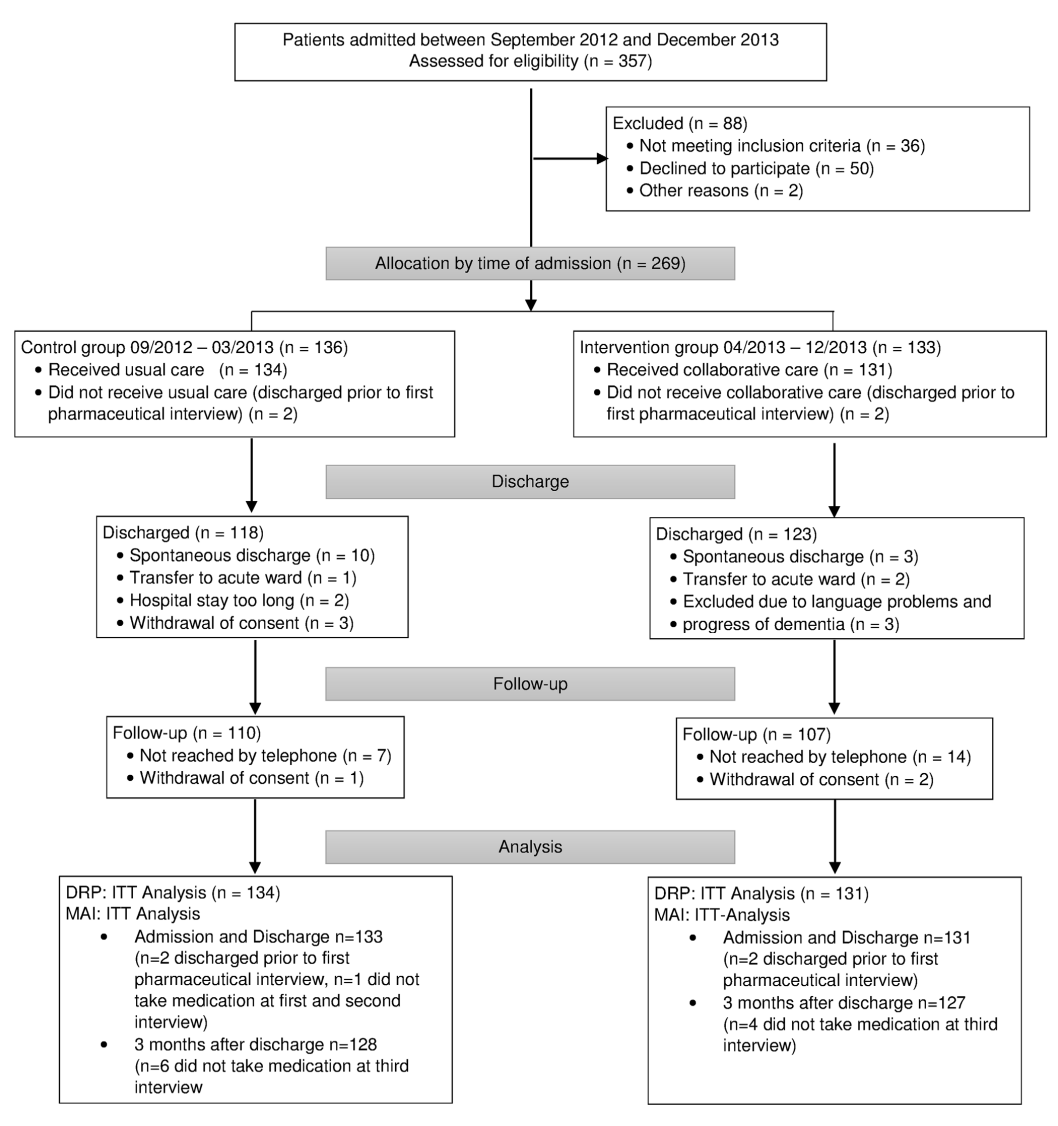
Flow diagram. ITT, Intention to treat; DRP, Drug-Related Problems; MAI, Medication Appropriateness Index.

Depending on their time of admission, 269 patients were allocated in the control group (09/2012-03/2013) or intervention group (05/2013-12/2013). Prior to the first pharmaceutical interview four patients were discharged and were excluded from the analysis. The second pharmaceutical interview at discharge was completed by 241 (89.6%) patients. The follow-up period terminated in July 2014 with the last patient’s follow-up interview. The study protocol was completed by 217 (80.7%) patients that were reached by telephone at the follow-up. At this time, ten (six control, four intervention) patients no longer took medications. One patient’s psychiatric drug was discontinued during the hospital stay. Thus, the evaluation of their MAI scores was not possible. For the analysis of the number of unsolved DRP, all patients who completed the first interview were included (Fig 1).

Gender, age and number of comorbidities significantly differed at baseline between the control and intervention groups. Consequently, the analysis of the primary outcomes were adjusted for these variables via regression models. The other demographic data were similar between the groups. The baseline assessment of the MAI score was calculated via the summation of the weighted MAI scores, which were assessed with the scoring system of Samsa et al [28] of every drug the patient regularly took at admission; there were no significant differences (Table 1).

**Table 1:** Demographic data of the study population (n = 265).

Variable	Control (n = 134)	Intervention (n = 131)	p-value
**Gender** [n, (%)], female	43 (32.1%)	61 (46.6%)	0.016^a^
**Age** [mean years (± SD)]	45.2 (14.8)	49.1 (15.3)	0.038^b^
**Prior psychiatric hospitalizations** [n, (%)]			
0–1	74 (55.2%)	77 (58.8%)	
≥ 2	60 (44.8%)	54 (41.2%)	
			0.559^a^
**Number of drugs** [median, IQR]			
at admission	4 (2–6)	3 (2–5)	0.722^c^
at discharge	4 (2–6)	4 (2–6)	0.840^c^
three months after discharge	4 (2–6)	3 (2–6)	0.525^c^
**Psychiatric Diagnosis** [n, (%)]			
Mood [affective] disorder (F30-F39)	89 (65.4%)	88 (66.2%)	
Neurotic, stress-related and somatoform disorders (F40-F48)	21 (15.4%)	26 (19.5%)	
Schizophrenia, schizotypal and delusional disorders (F20-F29)	13 (9.6%)	9 (6.8%)	
Mental and behavioral disorders due to psychoactive substance use (F10-F19)	7 (5.1%)	5 (3.8%)	
Others	4 (2.9%)	3 (2.3%)	
			0.797^d^
**Number of comorbidities** [median, (IQR)]	2 (1–4)	3 (1–5)	0.005^c^
**Length of hospital stay** [median, (IQR)]	29.0 (19.8–47.3)	35.0 (22.0–49.0)	0.108^c^
**Patient MAI score at admission** [mean, (± SD)]^ϕ^	2.4 (3.5)	2.3 (3.5)	0.329^c^

^a^Chi-square-test

^b^Student’s t-test

^c^Mann-Whitney-U-test

^d^Fischer’s exact test

SD, standard deviation; IQR, interquartile range; MAI, Medication Appropriateness Index.

^ϕ^The MAI score could not been determined in one patient in the control group because he did not take drugs at the time of the first pharmaceutical interview.

### Primary Outcomes

**Fig 2:**
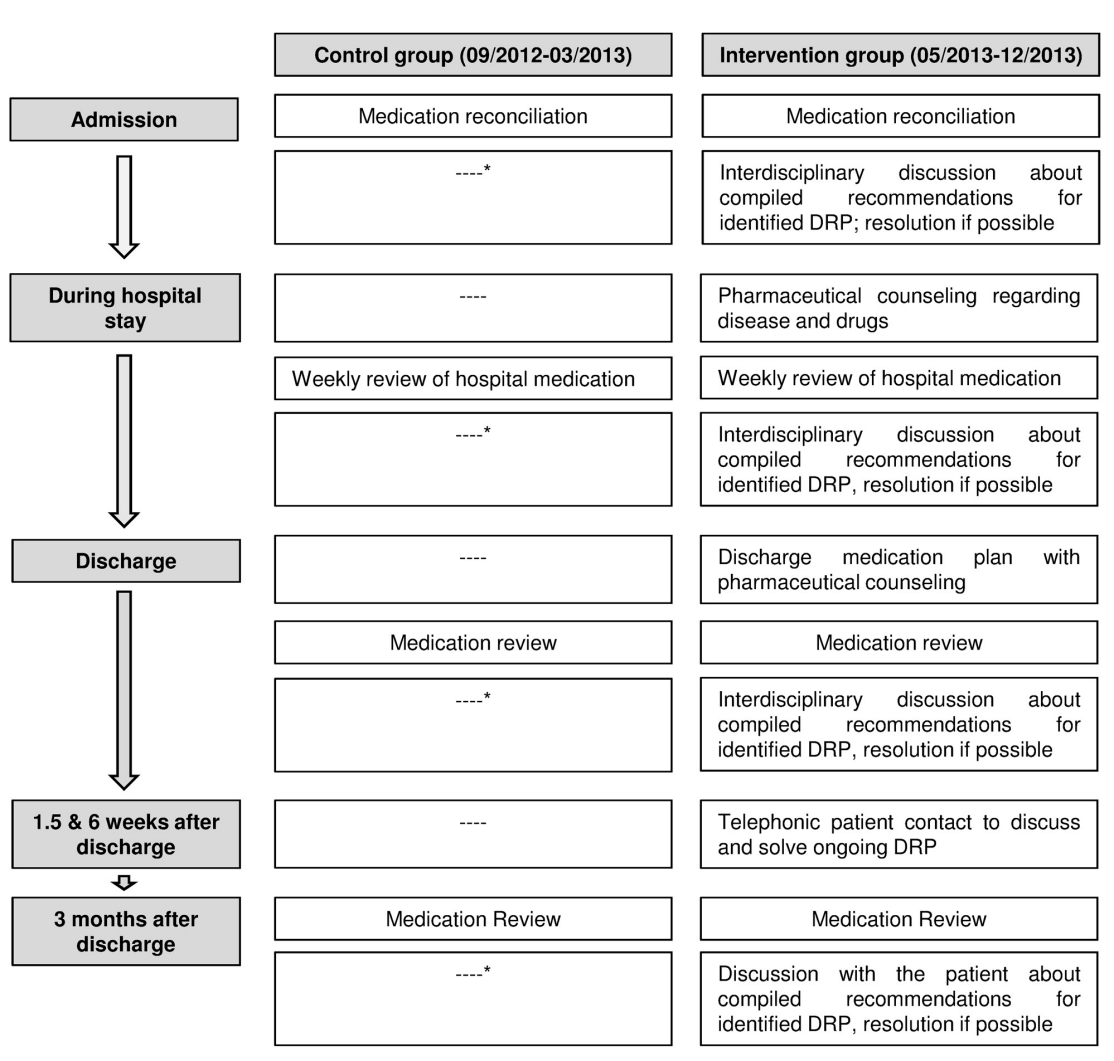
Procedure of pharmaceutical interventions. *Recommendations for identified DRP of patients in the control group were only disseminated to medical staff if they were serious or life threatening. DRP, Drug-Related Problems.

The change in the MAI score and the number of unsolved DRP were chosen as primary outcomes to assess the effect of the pharmaceutical interventions. The procedure of the pharmaceutical interventions is summarized in Fig 2.

MAI. The appropriateness of therapy was significantly improved in the intervention group compared with the control group from admission through discharge to follow-up, which correlates with the decrease in the patient MAI scores. An average of 2.3 points (SD: 3.5) in the patient MAI score at admission was reduced to 1.0 (SD: 2.0) at discharge and 0.8 (SD: 1.6) at follow-up in the intervention group. In the patients in the control group, the MAI score slightly decreased from admission (2.4 points, SD: 3.5) through discharge (2.4 points, SD: 3.0) to follow-up (2.2 points, SD: 2.8). The change in the patient MAI score from admission to discharge or follow-up is shown in Table 2. A negative score reflects an enhancement in the appropriateness of therapy, whereas a positive score indicates a deterioration. The adjusted effect of the intervention on the patient MAI score was an improvement of 1.4 points (95% CI: 0.8–2.1, p < 0.001) at discharge, which remained at follow-up (1.2 points, 95% CI: 0.6–1.9, p < 0.001).

**Table 2:** Primary Outcomes.

	Control (n = 134)	Intervention (n = 131)	Adj. effect size*	95% CI
**Change in summated MAI Score from admission to**				
discharge [mean, (SD)] ^ϕ^	0.0 (2.3)	-1.3 (3.0)	1.4	0.8–2.1
follow up [mean, (SD)] ^ϕϕ^	-0.4 (2.8)	-1.4 (2.8)	1.2	0.6–1.9
**Number of DRP**				
identified per patient [mean, (SD)]	3.1 (2.6)	3.0 (2.7)		
that remained unsolved per patient** [mean (SD)]	2.3 (2.1)	0.4 (0.9)	1.8	1.5–2.1

MAI, Medication Appropriateness Index; DRP, Drug-Related Problems; SD, standard deviation; CI, confidence intervals.

^ϕ^The MAI score could not be calculated in one patient in the control group because he did not take drugs at the time of the first and second interview.

^ϕϕ^ The MAI score could not be calculated in six and four patients in the control and intervention groups because they did not take drugs at the follow-up.

*The adjusted (adj.) effect size was computed from the treatment variable (in the case of the MAI, the interaction of treatment and time category) in the corresponding regression models and is thus adjusted for age, gender, comorbidities, number of drugs and length of hospital stays.

**after completing the study protocol

DRP. In total, 419 and 396 DRP were identified in 134 and 131 patients in the control and intervention groups, respectively, (p = 0.487, Mann-Whitney-U-test) in the comprehensive medication reviews of medical records at admission, discharge and weekly during the hospital stay. Of the identified DRP, 10.7 and 13.6% were rated as non-preventable ADE in the control and intervention groups, respectively. As shown in Table 3, the numbers of detected DRP that had the potential for (potential ADE, approximately 40% of all identified DRP) or actually contributed to patient harm (preventable ADE, approximately 3% of all detected DRP) were comparable between the groups. Both the preventable and non-preventable ADE were primarily resolved during the intervention in contrast with the control group. Significantly more DRP were not solved in the control (303 DRP) compared with the intervention patients (50 DRP) after the study protocol completion (Table 2). The adjusted effect of the intervention on the number of unsolved DRP after the entire study period was 1.8 (95% CI: 1.5–2.1, p < 0.001) less unsolved DRP per patient compared with usual care. In addition, the number of DRP per 1,000 patient-days was also evaluated for comparison with other medication safety studies in psychiatry, in which this denominator has commonly been used. The 4,811 and 5,149 patient-days in the control and intervention groups comply with 87.1 and 63.0 DRP per 1000 patient-days. In the control group, 76.9 DRP were not resolved compared with 5.8 DRP in the intervention patients.

**Table 3:** The potential and actual risk for patient harm of the identified DRP.

DRP, total number	Control		Intervention	
	Identified (n = 419)	Unsolved (%*) (n = 303)	Identified (n = 396)	Unsolved (%*) (n = 50)
little or no potential for harm	107	93 (83.9)	99	17 (17.2)
potential ADE^a^	252	182 (72.2)	231	28 (12.1)
preventable ADE^b^	15	12 (80.0)	12	2 (16.7)
Non-preventable ADE^c^	45	16 (35.6)	54	3 (5.6)

DRP, Drug-Related Problems; ADE, Adverse Drug Events

*of identified numbers.

^a^an error that had the potential for patient harm but did not contribute to patient harm during the study period

^b^patient harm occurred and was associated with a medication error

^c^patient harm occurred but was not associated with a medication error.

### Secondary Outcomes

The relevance of the identified DRP was typically estimated as minor (43.8%) or moderate (46.9%) in both groups. 45 and 30 major DRP occurred in the control and intervention phases, respectively. 12 identified DRP in the control patients were reported to the ward staff and/or patients by the pharmacist because of the potential to cause serious harm. Examples for DRP that were communicated to the physicians within the control phase were the concomitant prescription of omeprazole and clopidogrel, a patient with major depression that was not treated with an antidepressant or the concomitant use of two benzodiazepines, mirtazapine and risperidone in a patient that was fallen due to this combination.

Psychotropic drugs accounted for 54.4% of all detected DRP. Considering their prescribing frequency at admission, the potential to cause DRP of both psychotropic and non-psychotropic drugs were similar with 0.7 DRP per prescribed drug. The potentials of the 10 most commonly applied drugs to cause DRP were calculated and are illustrated in Table 4. Quetiapin most frequently caused DRP with 0.9 DRP per number of prescriptions, including potential drug-drug interactions, an inadequate application time or symptoms of an ADE. A reduction in the number of drug prescriptions with an increased potential to induce DRP, such as Imipramine and Carbamazepine, can be assumed in the intervention patients compared with the control. Imipramine and Carbamazepine induced 10 and 7 errors and were applied five and three times in the control; however, these drugs were not prescribed in the intervention group.

**Table 4:** Potential to cause DRP of the 10 most commonly prescribed drugs.

Drug	Potential to cause DRP*	Caused DRP (n = 815)	Prescriptions at admission (n = 604)
**Quetiapine**	0.9	56	65
**Ramipril**	0.8	27	33
**Escitalopram**	0.7	20	29
**Venlafaxine**	0.7	35	54
**Pantoprazole**	0.6	35	59
**Duloxetine**	0.6	17	30
**Mirtazapine**	0.5	36	67
**Levothyroxine**	0.5	25	46
**Lorazepam**	0.4	14	35
**Agomelatine**	0.4	12	30

DRP, Drug-Related Problems

*The potential was calculated by dividing the number of caused DRP by the number of prescriptions at admission.

The type of DRP and their outcomes were categorized according to the PIO classification system [30]. The problem categories “complex therapy regimen” (n = 77 of 815 detected DRP), “inadequate dosing frequency” (n = 67), “symptoms of an ADE” (n = 62) and “no or inadequate monitoring of drugs” (n = 60) commonly occurred in both groups. The majority of problems that frequently occurred but were not elucidated in the control patients were solved in the intervention group (Table 5). The outcomes of the solved DRP were predominately categorized as “improving patients’ adherence/satisfaction” or “increasing patients’ safety” (155 and 115 of 346 implemented interventions). 16% of the 346 implemented interventions were classified as “enhancing effectiveness of drug therapy”, whereas only 2% were classified as “reducing medication costs”. 13 interventions resulted in negative outcomes that led to a reversion of the intervention. Most of the interventions that caused negative outcome were related to the change, dose reduction or discontinuation of a drug (e.g. Mirtazapin) because of side effects, which led to a deterioration of the patients’ symptoms (e.g. worse sleep) and made a represcreption of the drug necessary. Collectively, the pharmaceutical recommendations were highly accepted by the ward staff (88.6% of all recommendations).

**Table 5:** DRP classification according to the PIO system^ϕ^.

	Control		Intervention	
Problem	Detected* (n = 419)	Solved* (%**) (n = 116)	Detected* (n = 396)	Solved* (%**) (n = 346)
Complex therapy regimen	37	4 (10.8)	40	35 (87.5)
Inadequate dosing frequency	47	4 (8.5)	20	14 (70.0)
Symptoms of an ADE	31	15 (48.4)	31	28 (90.3)
No or inadequate TDM	25	6 (24.0)	35	34 (97.1)
Insufficient or untreated indication	28	18 (64.3)	32	28 (87.5)
Dosage too low	35	12 (34.3)	24	19 (79.2)
No indication for the drug treatment	20	8 (40.0)	25	24 (96.0)
Inadequate duration of drug treatment	24	5 (20.8)	18	16 (88.9)
Discontinuation of drug due to ADE	14	14 (100.0)	23	23 (100.0)
Potential clinically relevant drug-drug interactions	18	6 (33.3)	18	14 (77.8)
Inadequate time of application	22	7 (31.8)	13	11 (84.6)
Did not use prescribed drugs	23	2 (8.7)	11	7 (63.6)
Transmission error	17	1 (5.9)	15	14 (93.3)
Clarification of drug dose	15	2 (13.3)	16	16 (93.3)
Drug dose too high	10	1 (10.0)	7	7 (100.0)

^ϕ^of the 15 most commonly detected problems during hospital stay.

PIO, Problem-Intervention-Outcome; ADE, Adverse Drug Event; TDM, Therapeutic Drug Monitoring

*total number of DRP

**percentage of detected problems

## Discussion

To our knowledge, this is the first study in psychiatric patients to provide evidence regarding the impact of a structured, interdisciplinary medicines management on the appropriateness of therapy and the number of DRP as indicators of medication safety compared with usual psychiatric care. Here, the MAI was first used as a valid tool to assess the therapy appropriateness in a psychiatric setting.

The trial identified a significant improvement in the appropriateness of therapy, which was sustained after discharge, and substantially less unsolved DRP as a result of our structured, interdisciplinary medication reviews with the subsequent implementation of changes in the therapeutic regimen. Problems that had the potential to cause harm were solved. Common sources of errors were identified and disseminated to the ward staff.

Attempts to improve medication safety are typically aimed at the identification and resolution of DRP and the enhancement in drug prescription [20,21]. Therefore, our first primary endpoint was the appropriateness of drug prescribing, which was assessed with the MAI. Prior research that addressed the impact of pharmaceutical interventions on the appropriateness of drug prescription was extended with this study because an evaluation in a psychiatric setting or a study in non-geriatric patients did not exist. As demonstrated for geriatric patients in several medical conditions [35–39], the intervention in our study significantly improved the appropriateness of the therapy measured by the MAI in middle aged, psychiatric patients. In contrast to these studies, the patient MAI scores at baseline were low in our study (2.3 versus 8.5 points in a trial conducted by Gillespie et al) [37]. A younger study population with half as many drugs prescribed per patient (49 versus 83 years and 3 versus 8 drugs per patient in the intervention group) contributed to this finding. Additionally, Schmader et al [40] indicated lower MAI scores for CNS drugs (1.2, SD 1.9 points) because of their classification as high risk medications compared with other drug groups. Nevertheless, the interdisciplinary approach evaluated in our study decreased the patient MAI score by 54.8%, which exceeded the decline of 41.2% identified by Gillespie et al [37]. In our trial, the enhanced appropriateness of therapy was also observed three months after discharge, which represents the sustainability of the intervention and the benefit of pharmaceutical patient contacts after discharge. Improvements in the appropriateness of prescriptions can be associated with fewer ADE [35] and improved outcomes [41], as well as decreased hospital revisits and total costs [42] in geriatric patients. However, the evaluation of these outcomes was beyond the scope of our study. Although these outcomes are considered transferable to non-geriatric psychiatric inpatients, no confirmatory data is available. Future studies should address this missing link.

Other important factors in terms of drug therapy safety are the identification and the resolution of DRP [21]. As confirmed in our study, Grasso et al [8] and Rothschild et al [5] verified the effectiveness of a medication review in the identification of DRP in psychiatric inpatients. Additionally, the patient interviews supported the identification of DRP, which was also ascertained by Viktil et al [43]. It can be assumed that the values of DRP were lower in the evaluation of Rothschild et al [5] (20.6 versus 81.8 DRP per 1,000 patient-days in our evaluation) because they did not interview the patients. Furthermore, the group excluded DRP with little or no potential for harm in contrast to our study, which included and addressed these errors. Errors with little or no potential for harm, such as an inadequate dosing frequency and a complex therapy, are likely to reduce patient adherence and are therefore worthy of identification, resolution and prevention [44].

To minimize the risk of medication-related harm for psychiatric patients, Rothschild et al [5] claimed the need for studies that evaluate strategies to reduce DRP caused by psychiatric and non-psychiatric drugs. The highly significant reduction in the number of unsolved DRP first emphasized the benefit of a strategy that implements pharmacist-led medication reviews with subsequent collaborative discussion of identified DRP in psychiatric wards to improve the drug therapy safety of psychiatric inpatients in a controlled trial. The primary outcome measure that was applied in our study, the number of unsolved DRP after the entire study period, has not been previously used in other controlled studies that assessed the benefit of a collaborative care model. The strength of the measure is the opportunity to assess the added value of the inter-professional approach to solve all DRP that occurred within the hospital stay compared with usual care. The percent of solved DRP compared with the control group was substantially higher (86.3% solved DRP compared with 27.9%). A study in internal medicine demonstrated a similar extensive percentage of solved DRP (60.7% of an average of 9.9 detected DRP per patient) with a comparable pharmaceutical care program. However, the percentage of solved DRP for control patients were not provided [45]. Nevertheless, these results likely indicate that the advantage of a structured pharmaceutical care program including medication reviews is independent of the medical discipline. However, the impact of 1.8 less unsolved DRP per patient in the intervention group on clinical outcomes, such as length of hospital stay or rate of rehospitalization, remains uncertain. Gillespie et al [46] although ascertained that the number of necessary hospitalizations was decreased by the resolution of 75% of identified DRP in geriatric patients, the transferability to middle-aged psychiatric patients is questionable. Following studies in psychiatry that focus on clinical outcomes are therefore needed.

A major limitation of our study is that it was designed as a non-randomized but consecutive trial. Assistant physicians alternated during the study period. We cannot rule out that the improvement in prescribing and the decrease in unsolved DRP was caused by the change of ward staff. Nevertheless, the consecutive design attempted to avoid a knowledge bias in the control group when recommendations for the resolution of DRP in the intervention patients on the same ward were compiled and discussed with the attending physicians. For the first investigation to assess the impact of pharmacist-led medication reviews on therapy appropriateness and the number of unsolved DRP in a controlled clinical trial, we chose to abstain from the randomized trial to minimize confounding. Moreover, the applied design did not ensure a comparable structure of patient characteristics at baseline; thus, the analysis of the primary outcome was adjusted for significant differences via a regression model.

A one-month washout period between study phases were chosen based on the stated average length of patient stay in the hospital of 21 days. However, this mean length of stay comprised also patient, which stayed only for one or two days in hospital to obtain diagnostic tests for example to detect a dementia. As a hospital stay of less than seven days was an exclusion criterion of our trial, the mean length of stay of our study population was longer with a median of 29.0 and 35.0 days in control and intervention group, respectively. Two patients of the control group were still present at ward for three and seven days, respectively when intervention phase started at 1 May 2014. However, the overlap did not affect the results because the first pharmaceutical interview at admission in the intervention phase was performed on 8 May and therefore after discharge of these two control patients.

The scoring of the MAI and the number of identified and unsolved DRP were not assessed by an independent rater, but by the two pharmacists who performed the medication reviews on the wards and recommended changes in the therapy. To avoid an over-estimation of the effect of the intervention, the MAI scoring, the classification and the relevance of the DRP were verified by a senior psychiatrist. However, an inter-rater-reliability-score between the pharmacists and the psychiatrist was not assessed. This second limitation will be addressed in a following evaluation when independent raters retrospectively assess the recorded therapy regimen.

As a result of the exploratory nature of our study, indirect measures were applied to assess the impact of an interdisciplinary medicines management in psychiatry. Because a significant benefit was verified, further evaluations should consider the analysis of more direct measurements for patient outcomes, such as the length of hospital stay or readmission rates.

Apart from this, the MAI does not address all issues of inappropriate prescribing. Under-prescription, side effects or compliance were not evaluated with the items of the MAI. Therefore, the calculated MAI score does not precisely reflect the appropriateness of therapy. Nevertheless, the MAI with its ten implicit criteria were chosen over other measures of inappropriate prescribing (e.g. Beers criteria, STOPP/START criteria) that employ explicit criteria. When using explicit criteria individual patient preferences and clinical and patient individual knowledge of the prescriber cannot be recognized. Additionally, other measures of inappropriate prescribing such as Beers criteria and STOPP/START criteria were solely developed for the assessment of appropriate therapy in older adults. However, the implicit character of the items of the MAI also allow its use in a younger study population, as we expected to have in our study. Another limitation is the assessment of the MAI score three months after discharge. The calculation of the MAI score based on the medication plan that was verified by the pharmacists in collaboration with the patient by telephonic consultation. However, the pharmacists did not have any other information from community physicians or pharmacies. Therefore, it is possible that the medication plan was incomplete or information were missing.

Only two non-acute wards of the same university hospital in Germany were studied. Thus, the generalizability of the results is limited. Additionally, it would have been favorable to estimate the time that was needed for the discussion with the medical team to assess its impact on the workload of the pharmacists. Future studies should focus on this topic. Finally, it was beyond the scope of our evaluation to assess the costs of DRP or additional services (for example, pharmaceutical medication review). However, the economic aspect is an important factor in the consideration of the permanent implementation of a new care model in the daily clinical routine. Therefore, an economic calculation will also be performed in a subsequent study.

In brief, despite several limitations, the structured pharmacist-led medication reviews with subsequent interdisciplinary discussion of DRP has been proven to be an effective tool to identify and solve DRP and therefore enhance the appropriateness of therapy in psychiatric inpatients. Thus, the permanent implementation of the interdisciplinary pharmaceutical care model in psychiatric hospitals appears to be a worthy strategy to improve medication safety in psychiatric patients. The promising results of this trial warrant further research that evaluates the impact of collaborative pharmaceutical care programs regarding direct clinical outcomes and health-related costs in psychiatry.

## Supporting Information

S1 CONSORT Checklist CONSORT Statement. (PDF)

S1 File Related manuscript by Pauly et al. (PDF)

S1 Protocol Study protocol German. (PDF)

S2 Protocol Study protocol English. (PDF)
