# Supplementary material for: Pharmacist-Led Medication Reviews to Identify and Collaboratively Resolve Drug-Related Problems in Psychiatry – A Controlled, Clinical Trial
Source: PLoS One. 2015 Nov 6;10(11):e0142011. doi: 10.1371/journal.pone.0142011 (PMC4636233; doi:10.1371/journal.pone.0142011)
Supplement: S1 File — (PDF) [file pone.0142011.s002.pdf]

Effect of a multi-dimensional and inter-sectoral intervention on the adherence of psychiatric patients

Anne Pauly[1], Carolin Wolf[1], Andreas Mayr[3], Bernd Lenz[2], Johannes Kornhuber[2], Kristina Friedland[1*]

[1]Molecular & Clinical Pharmacy, Friedrich-Alexander University Erlangen-Nürnberg (FAU), Erlangen, Germany

[2]Department of Psychiatry and Psychotherapy, Friedrich-Alexander University Erlangen-Nürnberg (FAU), Erlangen, Germany

[3]Department of Medical Informatics, Biometry and Epidemiology, Friedrich-Alexander-University Erlangen-Nürnberg (FAU), Erlangen, Germany

[*]Corresponding author:

Kristina.Leuner@fau.de

Abstract

In psychiatry, hospital stays and transitions to the ambulatory sector are susceptible to major changes in drug therapy that lead to complex medication regimens and common non-adherence among psychiatric patients. A multi-dimensional and inter-sectoral intervention is hypothesized to improve the adherence of psychiatric patients to their pharmacotherapy.

269 patients from a German university hospital were included in a prospective, open, clinical trial with consecutive control and intervention groups. Control patients (09/2012-03/2013) received usual care, whereas intervention patients (05/2013-12/2013) underwent a program to enhance adherence during their stay and up to three months after discharge. The program consisted of therapy simplification and individualized patient education (multi-dimensional component) during the stay and at discharge, as well as subsequent phone calls after discharge (inter-sectoral component). Adherence was measured by the "Medication Adherence Report Scale" (MARS) and the "Drug Attitude Inventory" (DAI).

The improvement in the MARS score between admission and three months after discharge was 1.33 points (95% CI: 0.73-1.93) higher in the intervention group compared to controls. In addition, the DAI score improved 1.93 points (95% CI: 1.15-2.72) more for intervention patients.

These two findings indicate significantly higher medication adherence following the investigated multi-dimensional and inter-sectoral program.

Introduction

Non-adherence to medication is one of the main causes for unsuccessful pharmacotherapy in psychiatry [1]. For example, the risk of hospitalization for non-adherent schizophrenic patients is reported to be four times higher than for adherent patients [2]. It is assumed that increasing adherence might be the most efficacious way to further improve health care outcome [3].

Adherence is influenced by social and economic factors, the health care team/system, the characteristics of the disease, disease therapies and patient-related factors, as defined by the WHO [3]. Successful interventions should ideally target several of these dimensions because single-faceted interventions have frequently failed to show an impact [4]. Besides affecting practical barriers to adherence (e.g. dosing frequency), perceptual barriers, including the patient's individual risk-benefit-assessment, should also be addressed [5]. Thus far, this dimension has only been considered in a few studies [5].

Successful research aimed at improving adherence in psychiatric patients and at developing a program for daily clinical practice in the hospital is rare. Previous investigations mainly enhanced the patient's knowledge and insight [6,7] and focused on outpatients [8,9], but did not combine the beneficial therapy simplifications [10] with individualized patient education. The hospital stay and the time after discharge are periods that are susceptible to major changes in drug therapy that lead to patient uncertainty [11]. Hence, the transition between inpatient care and the ambulatory sector should be a point of focus by health care professionals.

Therefore, we investigated the effect of a multi-dimensional and inter-sectoral program on the patients' adherence using a controlled study design in a psychiatric inpatient setting. The program started during the stay and ended three months after discharge. The additional program was administered only to patients in the intervention group. It included the provision of individualized verbal and written information regarding the patient's specific psychiatric medication and diseases with subsequent telephone calls after discharge that focused on the patients' concerns about their pharmacotherapy as well as maintaining adherence. Additionally, a medication review targeting the simplification of the pharmacotherapeutic regimen was performed. Control patients received usual care that included the

dispensing of medication by ward staff and group counseling about medication once during their stay. The patient's individual adherence was measured by the self-report questionnaires "Medication Adherence Report Scale" (MARS) [12] and "Drug Attitude Inventory" (DAI) [13] at admission, discharge and three months after discharge.

Materials and Methods

Trial design

The study was designed as a non-randomized, prospective and open trial with a control and subsequent intervention group. The consecutive design was chosen to avoid a "carry-over effect" of methods from the intervention to the control group. Therefore, a parallel design with randomization was not possible.

Setting and participants

The study was conducted on two open wards with a total of 44 beds at the Department of Psychiatry and Psychotherapy, University Hospital of Erlangen, Germany. Patients eligible for inclusion were ≥ 18 years who took at least one drug for a psychiatric condition, stayed a minimum of seven days on the ward and were able to communicate in German. Patients who met the inclusion criteria and gave written informed consent were assigned to the control or intervention group depending on the date of their admission. The control patients were included between September 2012 and March 2013 and the intervention patients between May and December 2013.

Ethics Statement

The Ethics Committee of the Friedrich-Alexander University Erlangen-Nuremberg (FAU) approved the study protocol on 26.07.2012. The trial was registered with the German Clinical Trials Register (DRKS00006358) and can be accessed via https://drks-neu.uniklinik-

freiburg.de. The study was registered after enrolment of patients started because the responsible persons weren't aware of the need to register before. No significant changes were made to the study protocol after approval of the ethical committee. The authors confirm that all ongoing and related trials for this intervention are registered.

Intervention

A multi-dimensional intervention was chosen due to its reported superiority to single-dimensional interventions [4,14,15]. The intervention was designed to create a program that addressed more than one of the five aforementioned factors influencing adherence [3]. The resulting multi-dimensional program focused on patient- and medication-related factors. Since, according to the published literature, [16–18] insight is one of the most important factors that improves adherence in mental health, the program was based on a psychoeducational intervention. However, because standardized psychoeducation alone is not always efficacious, the intervention was individualized and combined with a simplification of drug regimens [19]. Additionally, phone calls provided closure to the inter-sectoral gap.[20]

The multi-dimensional and inter-sectoral program included 1) medication management with a focus on a simplification of therapy regimen, 2) counseling of patients during the hospital stay with verbal and written information and a medication plan at discharge, 3) phone calls after discharge. As recommended in the literature [1,21,22], the intervention was personalized to meet the individual requirements of each patient. For example, the description of the side effects in the information leaflets was adjusted for every patient.

1) Weekly, two pharmacists on the ward conducted a chart review to assess reasonable simplifications of medication regimens. Simplifications included reducing dosing frequency when possible, discontinuing unnecessary drugs and adjusting the times that drugs were taken in order to achieve optimal efficacy, few side effects and a simple therapy

regimen. The simplifications were adjusted to the individual needs of each patient. In the intervention phase, these recommendations were discussed with the attending ward physicians who, together with the patient, decided the final implementation of the changes. For example, a common simplification concerned the once-daily dosage of Venlafaxine as a retard preparation that had been frequently administered twice daily prior to the intervention period.

In addition, the two pharmacists conducted a medication management to detect and solve drug-related problems, including drug-drug interactions or inappropriate dosing. Further information is provided elsewhere (unpublished data Wolf et al, 2014).

2) All intervention group patients received an individual consultation about their respective psychiatric disease and drugs. The patients were counseled about indication, mechanism of action, doses, side effects, interactions and instructions for use by a pharmacist. Additionally, the patients were informed about the delayed onset of effect and, if applicable, about the necessity to take the drug in order to prevent a relapse after recovery. Every patient received individualized written material based on the current state of the literature [23]. The duration of the consultation was adjusted based on the patient's prior knowledge and desire to ask questions.

At discharge, the pharmacists provided intensive discharge counseling including a medication plan with information that included the trade name, active component, indication, required dosage and dosing schedule as well as administering information. The structure of the medication plan was based on the planned standardized medication plan for Germany [24].

3) During the follow up after discharge, the pharmacists telephoned the intervention group patients twice. The calls occurred 1.5 weeks and 6 weeks after discharge and were aimed at motivating the patient to maintain the pharmacotherapy, answering questions and being informed about possible changes made by the primary care provider. If necessary, for

example, when a tendency toward suicidality was observed, a hospital psychiatrist was involved.

Outcome measures

The primary outcome was the change in adherence from baseline to follow-up three months after discharge as measured by the MARS [12]. The secondary outcome measure was the difference in attitude towards psychotropic drugs as determined by the DAI [13].

MARS

The MARS (Medication Adherence Report Scale) is a 5-item self-report questionnaire that asks about adherence in a continuous and sympathetic way [12]. It was translated into German, validated and considered reliable [13]. The MARS has been used to assess non-adherence over a variety of medical conditions including psychiatric diseases [25].

The patients score their own medication taking behavior on a Likert-scale ranging from ‘always’ (1 point), ‘often’, ‘sometimes’, ‘rarely’ to ‘never’ (5 points) in terms of the following parameters: forgetting medication, altering or omitting doses, taking less than instructed or discontinuing the medication. In the end, scores are summed up with a maximum of 25 and a minimum of 5. A higher score indicates more adherent behavior. To calculate a change score as a percentage, the maximal possible difference of 20 points is defined as 100%.

Item 1 of the MARS assesses unintentional non-adherence (forgetfulness), whereas items 2 – 5 determine intentional non-adherence such as deliberate altering of the dose or suspending the medications.

A cut-off value is not defined by the original initiators of the MARS. However, cut-off values between 20 and 24 have been used previously [25–27]. By applying a cut-off value to the MARS scores, the percentage of adherent patients can be determined. This can be advantageous

when comparing adherence rates measured by different methods. Due to the skewed distribution and its utilization in previous studies [28], a cut-off value of 24 was defined.

DAI

The Drug Attitude Inventory (DAI) [13] is a widely used and well-established self-rating instrument that assesses a patient's attitude towards psychotropic drugs. The DAI score is associated with the degree of non-adherence [29]. Although the DAI was primarily designed for antipsychotic drugs, it is by now also used for a variety of psychiatric diseases such as depression [30], thereby justifying its use in this trial.

The DAI originally contained 30 items, but a shortened version with 10 items that are considered to be predictive for the entire set, was developed. Both versions show good test-retest reliability and discriminative validity as well as a high internal consistency [31,32]. The DAI-10 is more frequently used due to its simplicity and equal psychometric properties.

The DAI consists of 10 statements by schizophrenic patients about their medications and includes subjective feelings as well as attitudes [13].

For every statement, the patients score whether it is true or false according to their own experiences with their drugs during the last four weeks. An answer indicating a positive attitude scores +1 point whereas an answer suggesting a negative attitude scores -1 point. A positive or negative sum score demonstrates a positive or negative attitude associated with (non-) adherence, respectively.

Collection of baseline and outcome data

Baseline data was collected after written informed consent of the patient and enrollment in the study. The pharmacists conducted a patient interview to assess demographic details, the therapy regimen and baseline adherence values measured by the MARS and DAI.

At discharge and three months after discharge (follow-up), the current medication profile for each patient was recorded and assessed by a pharmacist. The MARS and DAI were completed by the patients again. The data collected three months after discharge was usually obtained by telephone unless the patient wanted to return to the hospital to talk in person.

Statistical analysis

The sample size estimation was based on a study conducted by Finley et al that reported a 15% difference of adherence in control and intervention groups [33]. A minimum sample size of 123 patients was computed to detect a difference that was statistically significant at the 5% level with a power of at least 80% [34]. The study of Finley et al was chosen because it involved a similar program consisting of medication management, patient education and telephone follow-up [33].

Descriptive statistics are presented as the mean with standard deviation or the median with interquartile ranges for continuous variables. Categorical variables were presented as numbers and percentages. To check for group differences due to the allocation by time of admission, Chi-Square-Test or Fisher's-Exact-Test were applied for categorical variables, while Student's-t-Test and Mann-Whitney-U-Test were used for continuous variables.

The analysis of the primary and secondary outcomes was conducted following the intention-to-treat principle. Missing outcome values were imputed with the last valid observation of the patient (e.g., the baseline value). A sensitivity analysis showed that this imputation approach was more conservative than a corresponding analysis of the complete sample that resulted in higher effect sizes (results not shown).

For both endpoints (MARS and DAI), statistical regression analysis was performed to estimate the effect of the intervention while adjusting for group differences at baseline. The change of the outcome variables between baseline and follow-up served as response variables, while the

grouping variables, age, sex, comorbidities, number of medications at admission and the baseline score served as predictors. The estimated coefficient for the group variable therefore represents the adjusted effect of the intervention and was reported with a corresponding 95% CI. This procedure was repeated for both sum scores as well as for the single items.

Statistical analyses were performed using IBM SPSS Statistics for Windows Version 22.0 (SPSS Armonk, NY, USA: IBM Corp.) and the statistical programming environment R 3.0.2 (R Foundation for Statistical Computing, http://www.R-project.org/).

Results

Baseline characteristics

A total of 269 patients were included. 136 patients admitted between September 2012 and March 2013 and who met the inclusion criteria were allocated to the control group. Three patients were discharged prior to the first pharmaceutical interview and consequently were not included in the analyses of baseline and outcome variables. Recruitment for the intervention group began in April 2013 and ended in December 2013. 133 patients were included in the intervention group. Again, two patients were discharged early and therefore excluded from the analysis. Mostly due to spontaneous discharge, the discharge assessment was not completed for 23 patients (15 control and 8 intervention). 109 control and 107 intervention group patients were followed for three months after discharge. One patient did not take any medications between discharge and follow-up. Consequently, the MARS and DAI score were not assessed resulting in a total of 263 patients for analysis of the MARS. Six patients did not take psychiatric drugs during the last four weeks before follow-up. The DAI was not assessed for them at follow-up resulting in 258 evaluable questionnaires. All analyses were carried out following the intention-to-treat principle with imputed values, if necessary, for 263 patients and 258 patients, respectively. **(Fig. 1)**.

Fig. 1. Trial profile. ITT, Intention To Treat. Flow chart of control and intervention patients from allocation to group to analysis of date.

There were no significant differences between the two groups regarding demographic data such as marital and work status, nationality or living arrangements. Furthermore, the two groups did not differ significantly in terms of disease- and medication-related variables. However, patients in the intervention group were slightly older than the control patients. The intervention group consisted of a higher percentage of female patients and had more secondary diagnoses. These differences were considered in the statistical analysis. Mean MARS and DAI scores were similar in both groups at admission. **(Table 1)**

Table 1. Baseline characteristics of the study population.

	**Control (*n* = 133)**	**Intervention (*n* = 131)**	***p*-Value**
**Gender** (*n* women (%))	43 (32.33%)	61 (46.56%)	0.018[a]
**Age** (years (±SD))	45.44 (± 14.67)	49.10 (± 15.26)	0.048[b]
**Nationality** (*n* (%))			
German	120 (90.2%)	122 (93.1%)	
other	13 (9.8%)	9 (6.9%)	
			0.393[a]
**Living arrangements** (*n* (%))			
living with family	82 (61.7%)	89 (67.9%)	
living alone	47 (35.3%)	39 (29.8%)	
living in institution/sharing a flat	4 (3.0%)	3 (2.3%)	
			0.626[d]
**Marital status** (*n* (%))			
married	67 (50.4%)	68 (51.9%)	
single	44 (33.1%)	33 (25.2%)	
divorced	17 (12.8%)	22 (16.8%)	
widowed	5 (3.8%)	8 (6.1%)	
			0.405[d]
**Work status** (*n* (%))			
employee	37 (27.8%)	49 (37.4%)	
unemployed	25 (18.8%)	19 (14.5%)	
pensioners	21 (15.8%)	23 (17.6%)	
incapacitated	16 (12.0%)	14 (10.7%)	
self-employed	7 (5.3%)	6 (4.6%)	
official	7 (5.3%)	3 (2.3%)	
other	20 (15.0%)	17 (13.0.0%)	
			0.604[d]
**Education** (*n* (%))			
high-level	50 (37.6%)	44 (34.1%)	
intermediate-level	72 (54.1%)	74 (57.4%)	
low-level	7 (5.3%)	3 (2.3%)	
other	4 (3.0%)	8 (6.2%)	
			0.376[d]
**Prior psychiatric hospitalisations** (*n* (%))			
0-1	73 (54.9%)	77 (58.8 %)	
≥ 2	60 (45.1%)	54 (41.2 %)	
			0.523[a]
**Number of drugs** (Median (IQR))			

at admission	4 (2-6)	3 (2-5)	0.769[c]
at discharge	4 (2-6)	4 (2-6)	0.787[c]
3 months after discharge	4 (2-6)	3 (2-6)	0.577[c]
**Psychiatric Diagnosis** (*n* (%))			
Mood (affective) disorder (F30-F39)	89 (66.9%)	88 (67.2%)	
Neurotic, stress-related and somatoform disorders (F40-F48)	20 (15.0%)	26 (19.8%)	
Schizophrenia, schizotypal and delusional disorders (F20-F29)	13 (9.8%)	9 (6.9%)	
Mental and behavioral disorders due to psychoactive substance use (F10-F19)	7 (5.3%)	5 (3.8%)	
Others	4 (3.0%)	3 (2.3%)	
			0.747[d]
**Length of stay in days** (Median (IQR))	29.0 (20.0-47.5)	35.0 (22.0-49.0)	0.160[c]
**Number of comorbidities** (Median (IQR))	2.00 (1-4)	3.00 (2-5)	0.006[c]
**Baseline MARS** (Mean (SD))	22.23 (2.87)	22.02 (3.42)	0.919[c]
**Baseline DAI** (Mean (SD))	1.08 (3.45)	1.63 (3.75)	0.215[b]

IQR, Interquartile Range. DAI, Drug Attitude Inventory. MARS, Medication Adherence Report Scale. SD, Standard Deviation.

[a]Chi-square-test, [b]Student’s t-test, [c]Mann-Whitney-U-test, [d]Fisher’s exact test

Primary outcome measures

Self-reported adherence was measured by the MARS, which can range between 5 and 25 with a higher score indicating a more adherent behavior [12]. The mean (SD) MARS score of control patients increased during hospitalization from 22.23 (2.87) by 1.21 points (6.05%) to 23.44 (2.34). Three months after discharge, the MARS score decreased by 0.97 points (4.85%) near to its level at baseline, 22.47 (2.99). In the intervention group, the MARS score improved from 22.02 (3.42) at admission by 2.3 points (11.6%) to 24.34 (1.61) at discharge. This value declined by 0.59 points (2.95%) to 23.75 (2.08) at the follow-up. Considering the improvement of the MARS score from baseline to follow-up, the adjusted effect of the intervention was 1.33 points (95% CI 0.73 – 1.93). **(Fig. 2)** Importantly, 53.44% of the intervention group patients in comparison to 31.06% of patients in the control group achieved the possible maximum of 25 points in the MARS indicating a perfect adherent behavior. **(Fig. 3)**

Fig. 2. MARS scores. Development of MARS Scores with median and interquartile ranges for control and intervention group from baseline to follow-up three months after discharge. MARS, Medication Adherence Report Scale.

Fig. 3. Distribution of MARS scores at follow-up. Distribution of MARS scores in control and intervention group at follow-up three months after discharge. MARS, Medication Adherence Report Scale.

Intentional and non-intentional non-adherence were increased similarly. The adjusted effect size for improvement in non-intentional non-adherence (item 1) was 0.27 points (95% CI 0.05 – 0.49), for intentional non-adherence (items 2 – 5) 0.96 points (95% CI 0.39 – 1.51). **(Table 2)**

Table 2. Description of the individual items of the MARS.

	Control mean (SD)		Intervention mean (SD)			
	Baseline	Follow-up	Baseline	Follow-up	Adjusted effect*	95% CI
**MARS item**	**(*n* = 133)**	**(*n* = 132)**	**(*n* = 131)**	**(*n* = 131)**		
I forget to take them.	4.24 (0.87)	4.26 (0.88)	4.37 (0.80)	4.68 (0.57)	0.27	0.05 – 0.49
I alter the dose.	4.36 (0.87)	4.45 (0.79)	4.31 (0.88)	4.74 (0.59)	0.32	0.13 – 0.51
I stop taking them for a while.	4.65 (0.66)	4.67 (0.65)	4.52 (0.80)	4.84 (0.46)	0.26	0.11 – 0.42
I decide to miss out a dose.	4.52 (0.75)	4.58 (0.71)	4.47 (0.80)	4.78 (0.50)	0.21	0.06 – 0.36
I take less than instructed.	4.45 (0.77)	4.52 (0.76)	4.35 (0.84)	4.71 (0.61)	0.26	0.09 – 0.43
**Intentional Non-Adherence (Sum Score 2 – 5)**	**17.99 (2.72)**	**18.21 (2.70)**	**17.65 (3.12)**	**19.07 (2.04)**	**0.96**	**0.39 – 1.51**
**Sum Score MARS**	**22.23 (2.87)**	**22.47 (2.99)**	**22.02 (3.42)**	**23.75 (2.08)**	**1.33**	**0.73 – 1.93**

CI, Confidence Interval. MARS, Medication Adherence Report Scale. SD, Standard Deviation
*Estimated treatment effect from statistical regression model, adjusted for sex, age, comorbidities, number of medications at admission and the baseline MARS score.

Secondary outcome measures

Adherence in psychiatric patients is strongly affected by their attitude towards their medication [13]. Therefore, we used the DAI to investigate the self-reported attitude of our sample to further clarify the causes for non-adherence. The DAI ranges between -10 (negative attitude) and +10 (positive attitude) [13].

The mean (SD) DAI score increased in the control patients during their hospitalization from 1.08 (3.45) at baseline by 1.04 points (5.2%) to 2.12 (3.82) at discharge. After discharge, this value increased slightly until follow-up by 0.33 points (1.65%) to 2.45 (4.02). In the

intervention patients, the DAI score improved from 1.63 (3.75) at baseline by 2.79 points (13.95%) to 4.42 (3.35) at discharge and by 0.37 points (1.85%) to 4.79 (3.39) at follow-up. Conclusively, the adjusted effect of the intervention on the improvement of the DAI score to follow-up was 1.93 points (95% CI 1.15 – 2.72). At admission, 57.36% of the control group patients and 62.02% of the intervention group patients were classified as adherent (DAI > 0). In the control group, the percentage of patients regarded as adherent at discharge was 69.77% and three months after discharge, 72.09%. In contrast, the proportion of adherent patients in the intervention group increased to 87.60%, which was maintained until follow-up (=87.60%). **(Fig. 4)**

Fig. 4. Fraction of adherent patients measured by the DAI. Fraction of adherent patients in control group (open circle) and intervention group (filled circle) measured by the DAI. DAI, Drug Attitude Inventory.

Regarding the individual items of the DAI, an (adjusted) effect can be observed for items 3 ("I take medications of my free choice."), 6 ("I take medication only when I feel ill."), 7 ("I feel more normal on medication.") and 9 ("My thoughts are clearer on medication.") **(Table 3)**

Table 3. Description of the individual items of the DAI.

	**Control mean (SD)**		**Intervention mean (SD)**			
	**Baseline**	**Follow-up**	**Baseline**	**Follow-up**	**Adjusted effect***	**95% CI**
**DAI item**	**(*n* = 133)**	**(*n* = 129)**	**(*n* = 131)**	**(*n* = 129)**		
For me, the good things about medication outweigh the bad.	0.35 (0.52)	0.55 (0.56)	0.44 (0.56)	0.69 (0.44)	0.03	-0.11 – 0.40
I feel strange, „doped up", on medication.	0.36 (0.61)	0.59 (0.66)	0.54 (0.67)	0.76 (0.45)	0.24	-0.04 – 0.51
I take medications of my free choice.	0.66 (0.60)	0.76 (0.50)	0.60 (0.70)	0.93 (0.28)	0.24	0.06 – 0.41
Medications make me feel more relaxed.	0.75 (0.69)	0.18 (0.76)	-0.03 (0.72)	0.22 (0.72)	0.12	-0.08 – 0.32
Medications makes me feel tired and sluggish.	0.06 (0.66)	0.29 (0.69)	0.23 (0.68)	0.45 (0.64)	0.06	-0.12 – 0.24
I take medication only when I feel ill.	-0.16 (0.95)	0.03 (0.97)	-0.10 (0.96)	0.75 (0.64)	0.69	0.43 – 0.94
I feel more normal on medication.	-0.11 (0.74)	-0.11 (0.82)	-0.14 (0.83)	0.12 (0.86)	0.26	0.04 – 0.49
It is unnatural for my mind and body to be controlled by medications.	0.11 (0.79)	0.25 (0.82)	0.19 (0.82)	0.48 (0.74)	0.15	-0.06 – 0.36

My thoughts are clearer on medication.	-0.26 (0.71)	-0.28 (0.78)	-0.28 (0.78)	-0.05 (0.83)	0.26	0.07 – 0.46
Taking medication will prevent me from having a breakdown.	-0.19 (0.76)	0.19 (0.81)	0.19 (0.84)	0.43 (0.74)	0.08	-0.12 – 0.28
**Sum Score DAI**	**1.08 (3.45)**	**2.45 (4.02)**	**1.63 (3.75)**	**4.79 (3.39)**	**1.93**	**1.15 – 2.72**

CI, Confidence Interval. DAI, Drug Attitude Inventory. SD, Standard Deviation.
*Estimated treatment effect from statistical regression model, adjusted for sex, age, comorbidities, number of medications at admission and the baseline DAI score.

Discussion

To our knowledge, this is the first controlled study that assessed the impact of a multi-dimensional and inter-sectoral program during hospitalization and after discharge on the adherence of psychiatric patients. We used the period of hospitalization for adherence enhancing interventions. In addition, the patients received telephone calls after discharge.

The complex intervention chosen for this study enhanced both non-intentional (item 1 of the MARS) and intentional non-adherence (item 2 – 5 of the MARS). Although the difference between the control and intervention groups is highly significant at discharge and three months thereafter, it is striking that during hospitalization 26% of the intervention and 52% of the control patients were non-adherent. The literature suggests that about 23% of patients are at least partly non-adherent during hospitalization [35]. The high percentage in our study can be explained by the long duration of hospitalization of psychiatric patients e.g., 38.73 [29.25] days in our trial for the whole study population. It is conceivable that an extension or intensification of the interventions would achieve an even higher number of completely adherent patients.

The percentage of adherent patients at follow-up was considerably higher when considering the DAI (87.60% versus 53.44% measured by MARS). A recent study by Brain et al compared the DAI with an electronic Medication Event Monitoring System (MEMS) as an objective adherence measurement [36]. They found that the DAI demonstrated moderate sensitivity and low specificity that could lead to an over-estimation of the proportion of non-adherent patients. This could explain our observation of higher percentages compared to the MARS.

It was interesting to observe that the adherence of control patients was increased during the hospitalization – possibly by the usual interventions of the ward staff such as group sessions about medication – but decreased after discharge to its original level. This implies that usual adherence supporting interventions are not completely sustainable. The valuable hospitalization time should be used to institute enduring interventions akin to the program described and assessed in this trial.

The difference between the groups in the DAI's item 3 ("I take medications of my free choice") could suggest a higher sense of participation in the decision making process in the intervention patients, which has been reported to increase adherence [37]. The improvement in item 6 ("I take medication only when I feel ill") seems to demonstrate a better knowledge about drug instructions in the intervention patients, possibly a result of the counseling provided by the pharmacists. The differences in items 7 and 9 could be attributed to a better response to pharmacotherapy, however a clinical outcome that would confirm this suggestion was not assessed.

Though the MARS [12] and DAI [13] are available as valid and reliable instruments, controlled studies in psychiatry that employ them are rare. De Las Cuevas et al used the DAI in a sample of depressed patients [38]. Firstly, they used the Morisky questionnaire to divide the study population into adherers and non-adherers. Then, they assessed a mean DAI score of 4.92 for adherent patients, which is comparable to the score measured in our study (4.79 at follow-up). An observational study by Medina et al reported a mean DAI score of 2.1 at discharge in schizophrenic and bipolar patients [39], which corresponds to the DAI score of control patients (2.12) in our trial. Even though a high percentage achieved a DAI score > 0, the absolute scores of (non-) adherent patients remain rather low, between 2 and 5. This might indicate, in concordance with Brain et al, that the optimal DAI cut-off value to distinguish between adherers and non-adherers could be around 4 [36].

Canales et al conducted a controlled study with a similar interventional program in an American psychiatric inpatient setting, but did not follow patients after discharge [40]. They provided pharmacy services, including weekly medication reviews, pharmacotherapy recommendations and medication education. They did not assess adherence, but focused on several outcome parameters. Clinical response in the intervention patients was better, and there were less adverse effects. Even though the transferability of the results to the German health care setting and vice versa is not entirely possible, it can be assumed that the program's benefits would extend beyond adherence to include clinical outcomes.

There are several limitations concerning this study. First of all, the chosen method for measuring adherence was a self-report questionnaire. A patient’s self-report is an indirect and subjective approach, and thus a less valid way of measuring adherence compared to direct methods [10]. Nonetheless, self-report is widely used due to its cost-effectiveness and practicality [9]. Velligan et al even asserted that data from subjective measurements were best correlated with the clinical state, thereby justifying the use of the MARS [41]. The DAI is also reported to be a predictor of adherence measured objectively by MEMS [36].

Another principal limitation of the study is that it was designed as a non-randomized trial. Seasonal fluctuations and rotating physicians influence the patients’ behavior and lead to a possible over- as well as under-estimation of the intervention’s effect size.

The study time was too short for measuring relapse and subsequent readmission to the hospital [9]. However, we hypothesize reduced relapse and readmission rates following the intervention because more adherent behavior has been shown to relate to improved clinical outcome [42], including reduced rates of relapses and re-hospitalizations [43,44]. It was beyond the scope of our trial to determine the impact of the intervention on these outcomes. Because numerous interventions fail to change the adherence due to narrow or non-individualized interventions

[4], the results of this trial support a successful method that should be further investigated with respect to clinical and economic outcomes.

It would have been favorable to divide the study population into subgroups to determine the impact of the single interventions in comparison to the whole program. Since this was not carried out, the identification of the most efficacious component of the program is not possible. However, it has already been established that standardized psychoeducation alone is not effective for promoting adherence [14]. We speculate that the promising element of individualization in our psychoeducational program is a main factor underlying the observed effect.

In conclusion, it can be determined that the implementation of a multi-dimensional and inter-sectoral program enhances the patients' adherence significantly up to three months after discharge. Considering the strong need for successful interventions to improve adherence among psychiatric patients, further studies are needed to explore the impact on clinical and economic outcomes.

Acknowledgement

The authors thank the "Department of General Practice and Health Services Research and Department of Internal Medicine VI, Clinical Pharmacology and Pharmacoepidemiology, University Hospital Heidelberg, Heidelberg, Germany" for the provision of the German version of the MARS.

References

1. Chapman SCE, Horne R (2013) Medication nonadherence and psychiatry. Curr Opin Psychiatry 26: 446–452. Available: http://www.scopus.com/inward/record.url?eid=2-s2.0-84881377249&partnerID=tZOtx3y1. Accessed 1 April 2014.

2. Morken G, Widen JH, Grawe RW (2008) Non-adherence to antipsychotic medication, relapse and rehospitalisation in recent-onset schizophrenia. BMC Psychiatry 8: 32. Available: http://www.scopus.com/inward/record.url?eid=2-s2.0-44249114045&partnerID=tZOtx3y1. Accessed 26 November 2014.

3. Sabate E (2003) Adherence to long-term therapies: evidence for action. World Heal Organ. Available: http://www.who.int/chp/knowledge/publications/adherence_full_report.pdf?ua=1. Accessed 25 July 2014.

4. Haynes RB, Ackloo E, Sahota N, McDonald HP, Yao X (2008) Interventions for enhancing medication adherence. Cochrane database Syst Rev. Available: http://www.scopus.com/inward/record.url?eid=2-s2.0-44949190590&partnerID=tZOtx3y1. Accessed 13 July 2014.

5. Horne R, Weinman J, Barber N, Elliott R (2005) Concordance , adherence and compliance in medicine taking. Rep Natl Co-ord Cent NHS Serv Deliv Organ R D: 1–331.

6. Al-Saffar N, Deshmukh AA, Carter P, Adib SM (2005) Effect of information leaflets and counselling on antidepressant adherence: open randomised controlled trial in a psychiatric hospital in Kuwait. Int J Pharm Pract 13: 123–132. Available: http://www.scopus.com/inward/record.url?eid=2-s2.0-20444415664&partnerID=tZOtx3y1. Accessed 18 July 2014.

7. Finley PR, Rens HR, Pont JT, Gess SL, Louie C, et al. (2003) Impact of a collaborative care model on depression in a primary care setting: A randomized controlled trial. Pharmacotherapy 23: 1175–1185. Available: http://www.scopus.com/inward/record.url?eid=2-s2.0-0141456709&partnerID=40&md5=bcf231ac26d91d3b93b53d1fa1185de7.

8. Rubio-Valera M, Serrano-Blanco A, Magdalena-Belío J, Fernández A, García-Campayo J, et al. (2011) Effectiveness of pharmacist care in the improvement of adherence to antidepressants: a systematic review and meta-analysis. Ann Pharmacother 45: 39–48. Available: http://www.scopus.com/inward/record.url?eid=2-s2.0-78751548096&partnerID=tZOtx3y1. Accessed 17 July 2014.

9. Al-Jumah KA, Qureshi NA (2012) Impact of pharmacist interventions on patients' adherence to antidepressants and patient-reported outcomes: A systematic review. Patient Prefer Adherence 6: 87–100. Available: http://www.scopus.com/inward/record.url?eid=2-s2.0-84857088941&partnerID=40&md5=5a25bc5889e0584e4c794d7ad1c4b03d.

10. Osterberg L, Blaschke T (2005) Adherence to medication. N Engl J Med 353: 487–497. Available: http://www.scopus.com/inward/record.url?eid=2-s2.0-23044442340&partnerID=tZOtx3y1. Accessed 11 July 2014.

11. Viktil KK, Blix HS, Eek AK, Davies MN, Moger TA, et al. (2012) How are drug regimen changes during hospitalisation handled after discharge: a cohort study. BMJ Open 2. Available: http://www.scopus.com/inward/record.url?eid=2-s2.0-84873165480&partnerID=tZOtx3y1. Accessed 24 October 2014.

12. Horne R, Weinman J (1999) Patients' beliefs about prescribed medicines and their role in adherence to treatment in chronic physical illness. J Psychosom Res 47: 555–567. Available: http://www.scopus.com/inward/record.url?eid=2-s2.0-0033398683&partnerID=tZOtx3y1. Accessed 20 July 2014.

13. Hogan TP, Awad AG, Eastwood R (1983) A self-report scale predictive of drug compliance in schizophrenics: Reliability and discriminative validity. Psychol Med 13: 177–183. Available: http://www.scopus.com/inward/record.url?eid=2-s2.0-0020535524&partnerID=40&md5=40e0532c73331d98bb1eeebcb909452c.

14. Chong WW, Aslani P, Chen TF (2011) Effectiveness of interventions to improve antidepressant medication adherence: a systematic review. Int J Clin Pract 65: 954–975. Available: http://www.scopus.com/inward/record.url?eid=2-s2.0-80051887513&partnerID=tZOtx3y1. Accessed 18 July 2014.

15. Roter DL, Hall JA, Merisca R, Nordstrom B, Cretin D, et al. (1998) Effectiveness of Interventions to Improve Patient Compliance a Meta-Analysis. Med Care 36: 1138–1161. Available: http://www.scopus.com/inward/record.url?eid=2-s2.0-0032135104&partnerID=tZOtx3y1.

16. Julius RJ, Novitsky MA, Dubin WR (2009) Medication adherence: a review of the literature and implications for clinical practice. J Psychiatr Pract 15: 34–44. Available: http://www.scopus.com/inward/record.url?eid=2-s2.0-67650105447&partnerID=tZOtx3y1. Accessed 25 July 2014.

17. Kasper S, Saya L, Tekin B, Loze JY (2009) How to improve adherence to antipsychotic treatment: Outcomes of the IMproving PAtient outComes in psychiaTry (IMPACT) Berlin 2009 meeting workshop. Int J Psychiatry Clin Pract 13: 245–252. Available: http://www.scopus.com/inward/record.url?eid=2-s2.0-70350746378&partnerID=40&md5=a871232f16dc2758360d105b20dfa393.

18. Mohamed S, Rosenheck R, He H, Yuping N (2014) Insight and attitudes towards medication among inpatients with chronic schizophrenia in the US and China. Soc Psychiatry Psychiatr Epidemiol 49: 1063–1070. Available: http://www.scopus.com/inward/record.url?eid=2-s2.0-84903765807&partnerID=tZOtx3y1. Accessed 18 July 2014.

19. Farooq S, Naeem F (2014) Tackling nonadherence in psychiatric disorders: current opinion. Neuropsychiatr Dis Treat 10: 1069–1077. Available: http://www.scopus.com/inward/record.url?eid=2-s2.0-84902304759&partnerID=tZOtx3y1. Accessed 21 July 2014.

20. Mistiaen P, Poot E (2006) Telephone follow-up, initiated by a hospital-based health professional, for postdischarge problems in patients discharged from hospital to home. Cochrane database Syst Rev: CD004510. Available: http://www.scopus.com/inward/record.url?eid=2-s2.0-42749105095&partnerID=tZOtx3y1. Accessed 11 December 2014.

21. Zullig LL, Peterson ED, Bosworth HB (2013) Ingredients of successful interventions to improve medication adherence. JAMA 310: 2611–2612. Available: http://www.scopus.com/inward/record.url?eid=2-s2.0-84890905739&partnerID=tZOtx3y1. Accessed 11 April 2014.

22. Cutrona SL, Choudhry NK, Fischer MA, Servi A, Liberman JN, et al. (2010) Modes of delivery for interventions to improve cardiovascular medication adherence. Am J Manag Care

16: 929–942. Available: http://www.scopus.com/inward/record.url?eid=2-s2.0-78650957841&partnerID=tZOtx3y1.

23. Benkert O, Hippius H (2013) Compendium of psychiatric pharmacotherapy. Springer-Verlag London Ltd.

24. Aly AF (2013) Der einheitliche Medikationsplan: Standardisierte Informationsquelle für die Arzneimitteltherapie. Krankenhauspharmazie 34: 240–241. Available: http://www.scopus.com/inward/record.url?eid=2-s2.0-84878249698&partnerID=tZOtx3y1.

25. Bowskill R, Clatworthy J, Parham R, Rank T, Horne R (2007) Patients' perceptions of information received about medication prescribed for bipolar disorder: implications for informed choice. J Affect Disord 100: 253–257. Available: http://www.scopus.com/inward/record.url?eid=2-s2.0-34249281242&partnerID=tZOtx3y1. Accessed 26 March 2014.

26. Butler JA, Peveler RC, Roderick P, Horne R, Mason JC (2004) Measuring compliance with drug regimens after renal transplantation: comparison of self-report and clinician rating with electronic monitoring. Transplantation 77: 786–789. Available: http://www.scopus.com/inward/record.url?eid=2-s2.0-1642364925&partnerID=tZOtx3y1. Accessed 25 March 2014.

27. Clatworthy J, Price D, Ryan D, Haughney J, Horne R (2009) The value of self-report assessment of adherence, rhinitis and smoking in relation to asthma control. Prim Care Respir J 18: 300–305. Available: http://www.scopus.com/inward/record.url?eid=2-s2.0-70849100662&partnerID=tZOtx3y1. Accessed 21 July 2014.

28. Stange D, Kriston L, von-Wolff A, Baehr M, Dartsch DC (2013) Reducing cardiovascular medication complexity in a german university hospital: Effects of a structured pharmaceutical management intervention on adherence. J Manag Care Pharm 19: 396–407. Available: http://www.scopus.com/inward/record.url?eid=2-s2.0-84878983373&partnerID=tZOtx3y1.

29. Awad AG (1993) Subjective response to neuroleptics in schizophrenia. Schizophr Bull 19: 609–618. Available: http://www.scopus.com/inward/record.url?eid=2-s2.0-0027185944&partnerID=tZOtx3y1.

30. De Las Cuevas C, Sanz EJ (2007) Attitudes toward psychiatric drug treatment: the experience of being treated. Eur J Clin Pharmacol 63: 1063–1067. Available: http://www.scopus.com/inward/record.url?eid=2-s2.0-35248844992&partnerID=tZOtx3y1. Accessed 18 July 2014.

31. Hogan TP, Awad AG (1992) Subjective response to neuroleptics and outcome in schizophrenia: A re-examination comparing two measures. Psychol Med 22: 347–352. Available: http://www.scopus.com/inward/record.url?eid=2-s2.0-0026721876&partnerID=tZOtx3y1.

32. Nielsen RE, Lindström E, Nielsen J, Levander S (2012) DAI-10 is as good as DAI-30 in schizophrenia. Eur Neuropsychopharmacol 22: 747–750. Available: http://www.scopus.com/inward/record.url?eid=2-s2.0-84865565648&partnerID=tZOtx3y1. Accessed 7 April 2014.

33. Finley PR, Rens HR, Pont JT, Gess SL, Louie C, et al. (2002) Impact of a collaborative pharmacy practice model on the treatment of depression in primary care. Am J Heal Pharm 59:

1518–1526. Available: http://www.scopus.com/inward/record.url?eid=2-s2.0-0037101866&partnerID=40&md5=7ac9f53e720ea122931fb6be00891080.

34. Kranke P, Schuster F, Muellenbach R, Kranke EM, Roewer N, et al. (2008) Grundlagen und Prinzipien klinischer Studien: Wie viele Patienten sollen (müssen) untersucht werden? Fallzahlschätzung in klinischen Studien. Kardiotechnik 17: 114–117. Available: http://www.scopus.com/inward/record.url?eid=2-s2.0-57649136654&partnerID=tZOtx3y1.

35. Carow F, Rieger K, Walter-Sack I, Meyer M, Peters F, et al. (2012) Objective assessment of nonadherence and unknown co-medication in hospitalized patients. Eur J Clin Pharmacol 68: 1191–1199. Available: http://dx.doi.org/10.1007/s00228-012-1229-2.

36. Brain C, Allerby K, Sameby B, Quinlan P, Joas E, et al. (2013) Drug attitude and other predictors of medication adherence in schizophrenia: 12 months of electronic monitoring (MEMS®) in the Swedish COAST-study. Eur Neuropsychopharmacol 23: 1754–1762. Available: http://www.scopus.com/inward/record.url?eid=2-s2.0-84887608510&partnerID=tZOtx3y1. Accessed 7 April 2014.

37. Horne R (1993) One to be taken as directed: Reflections on non-adherence (non-compliance). J Soc Adm Pharm 10: 150–156. Available: http://www.scopus.com/inward/record.url?eid=2-s2.0-0027860353&partnerID=tZOtx3y1.

38. De las Cuevas C, Peñate W, Sanz EJ (2014) Risk factors for non-adherence to antidepressant treatment in patients with mood disorders. Eur J Clin Pharmacol 70: 89–98. Available: http://www.scopus.com/inward/record.url?eid=2-s2.0-84891846494&partnerID=tZOtx3y1. Accessed 7 April 2014.

39. Medina E, Salvà J, Ampudia R, Maurino J, Larumbe J (2012) Short-term clinical stability and lack of insight are associated with a negative attitude towards antipsychotic treatment at discharge in patients with schizophrenia and bipolar disorder. Patient Prefer Adherence 6: 623–629. Available: http://www.scopus.com/inward/record.url?eid=2-s2.0-84872827623&partnerID=tZOtx3y1. Accessed 8 May 2014.

40. Canales PL, Dorson PG, Crismon ML (2001) Outcomes assessment of clinical pharmacy services in a psychiatric inpatient setting. Am J Heal Pharm 58: 1309–1316. Available: http://www.scopus.com/inward/record.url?eid=2-s2.0-0035879363&partnerID=40&md5=8706768c34ffdb37b67a62d144cc516f.

41. Velligan DI, Wang M, Diamond P, Glahn DC, Castillo D, et al. (2007) Relationships among subjective and objective measures of adherence to oral antipsychotic medications. Psychiatr Serv 58: 1187–1192. Available: http://www.scopus.com/inward/record.url?eid=2-s2.0-34848817407&partnerID=tZOtx3y1. Accessed 24 July 2014.

42. Viswanathan M, Golin CE, Jones CD, Ashok M, Blalock SJ, et al. (2012) Interventions to Improve Adherence to Self-administered Medications for Chronic Diseases in the United States: A Systematic Review. Ann Intern Med. doi:10.7326/0003-4819-157-11-201212040-00538.

43. Geddes JR, Carney SM, Davies C, Furukawa TA, Kupfer DJ, et al. (2003) Relapse prevention with antidepressant drug treatment in depressive disorders: a systematic review. Lancet 361: 653–661. Available: http://www.scopus.com/inward/record.url?eid=2-s2.0-0037460745&partnerID=tZOtx3y1. Accessed 25 July 2014.

44. Masand P, Narasimhan M (2006) Improving Adherence to Antipsychotic Pharmacotherapy. Curr Clin Pharmacol 1: 47–56. Available: http://www.scopus.com/inward/record.url?eid=2-s2.0-34547551320&partnerID=tZOtx3y1. Accessed 28 July 2014.

Supporting Information

S1 File. Studienprotokoll.

S2 File. Study protocol.

S3 File. CONSORT Statement.
