# Supplementary material for: Pharmacist-Led Medication Reviews to Identify and Collaboratively Resolve Drug-Related Problems in Psychiatry – A Controlled, Clinical Trial
Source: PLoS One. 2015 Nov 6;10(11):e0142011. doi: 10.1371/journal.pone.0142011 (PMC4636233; doi:10.1371/journal.pone.0142011)
Supplement: S1 Protocol — (PDF) [file pone.0142011.s003.pdf]

Pharmazeutische Betreuung stationärer psychiatrischer Patienten - Studienprotokoll

Hintergrund

Die 12-Monats-Prävalenz psychiatrischer Erkrankungen liegt in Deutschland für Frauen bei 37% und für Männer bei 25,3% [4]. Sie hat in den letzten Jahren, vor allem für depressive Erkrankungen, zugenommen [5]. Eine genaue und vollständige Arzneimittelanamnese ist bei dieser Patientengruppe enorm wichtig, da psychiatrische Erkrankungen häufig von Komorbiditäten und damit von Polypharmazie begleitet werden [6]. Das Risiko für AbP steigt damit exponentiell an. Weiterhin ist durch die spezielle Wirkweise vieler psychotroper Medikamente (Wirkungseintritt erst nach 2-3 Wochen, Auftreten von Nebenwirkungen jedoch sofort) die Rate der Therapieabbrüche sehr hoch. Die Anzahl adhärenter Patienten schwankt auch nach Einführung der modernen Präparate zwischen 20-70% [2]. Die Adhärenz stellt jedoch ein sehr wichtiges Standbein einer adäquaten und erfolgreichen Therapie psychischer Erkrankungen dar. Das Risiko einer erneuten Krankenhauseinweisung ist nach Valenstein et al für einen nicht-adhärenten schizophrenen Patienten im Vergleich zu einem adhärenten schizophrenen Patienten um das 2,4fache erhöht [7]. Ähnliches gilt für Patienten mit affektiven Störungen. Ein vorzeitiger Therapieabbruch führt bei affektiven Störungen zu einer 77%igen Erhöhung des Rückfallrisikos [8].

Ein Ansatz zur Reduktion von AbP sowie zur Verbesserung der Adhärenz ist das Konzept der medizinisch-pharmazeutischen Betreuung. Medizinisch-pharmazeutische Betreuung basiert auf der intensiven Zusammenarbeit zwischen Patient, behandelndem Arzt und Apotheker. Ziel ist es, den Patienten gemeinsam mit dem behandelnden Arzt zu einer sicheren und sachgerechten Arzneimittelanwendung anzuleiten, mögliche AbP zu erkennen und zu lösen, die Arzneimitteltherapie zu optimieren und daraus resultierend die Lebensqualität des Patienten zu verbessern.

Eine niederländische Arbeitsgruppe um Joanna Klopotowska untersuchte den Einfluss eines Stationsapothekers auf die Anzahl AbP auf einer Intensivstation. Dabei konnte eine Reduktion der AbP von 190,5 pro 1000 beobachteten Patiententagen auf 62,5 pro 1000 beobachteten Patiententagen während der Interventionsphase nachgewiesen werden [9]. In einer Studie zur Pharmazeutischen Betreuung von Patienten mit koronarer Herzkrankheit nach aortokoronarer Bypassoperation am Universitätsklinikum Erlangen konnte eine signifikante Adhärenzsteigerung gezeigt werden. Nach 12 Monaten lag die Adhärenz in der Interventionsgruppe bei 90% (Basiswert: 77%) und in der Kontrollgruppe bei 71% (Basiswert 81%). Das Betreuungskonzept umfasste neben Gesprächen mit einem Krankenhausapotheker während des stationären Aufenthaltes auch Follow-up-Gespräche bei Ambulanzbesuchen und beinhaltete die Aushändigung schriftlicher Unterlagen [10].

Mit der vorliegenden geplanten Studie zur pharmazeutischen Betreuung von psychiatrischen Patienten auf Station, soll untersucht werden, ob sich ähnlich positive Effekte auf die Anzahl der AbP, sowie die Medikamentenadhärenz dieser Patientengruppe erzielen lassen. Zur Quantifizierung dienen hierbei unter anderem validierte Fragebögen.

Unseres Wissens nach gibt es in Deutschland bisher keine vergleichbaren Studien mit psychiatrischen Patienten. Ein erstes Projekt in Großbritannien erfasste 282 Interventionen durch den Apotheker auf Station [11]. Eine Betreuungsstudie in den USA brachte bei 62% der Patienten eine mehr als 30%ige

Verbesserung im Brief Psychiatric Rating Scale [12]. In einer weiteren amerikanischen Studie wurde eine Verbesserung der Adhärenz um 19% erreicht [13]. Mit der vorliegenden, geplanten Studie soll erstmals medizinisch-pharmazeutische Betreuung durch Stationsapotheker in enger Kooperation mit den Klinikärzten auf einer deutschen, psychiatrischen Station implementiert, aber auch gleichzeitig der Nutzen der geleisteten Betreuung des Apothekers quantitativ auswertbar dargestellt werden.

Zielsetzung des Projektes

Ziel des geplanten Projekts ist es, in einer klinischen Studie zu erfassen, ob pharmazeutische Betreuung durch Integration eines Stationsapotheker in das multidisziplinäre Behandlungsteam der Klinik für Psychiatrie und Psychotherapie des Universitätsklinikums Erlangen zu einer Reduktion bzw. Verbesserung folgender Parameter führen kann: der Anzahl Arzneimittel-bezogener Probleme (AbP), der Angemessenheit der Therapie und der Therapietreue, vor allem nach Entlassung.

Methoden

Bei der geplanten Studie zur pharmazeutischen Betreuung handelt es sich um eine prospektive, offene Interventionsstudie mit sequenziellem Kontrollgruppendesign.

Eingeschlossen werden psychiatrische Patienten der Stationen P21 und P31 der Psychiatrischen und Psychotherapeutischen Universitätsklinik Erlangen unabhängig ihrer Diagnose.

Weitere Einschlusskriterien müssen erfüllt sein:

- Alter: ≥ 18
- Fähigkeit, selbstständig Fragebögen auszufüllen
- Fähigkeit, gesprochenes und geschriebenes Deutsch zu verstehen
- Einwilligungsfähigkeit
- Stationärer Aufenthalt in der Psychiatrischen Klinik Erlangen > 7 Tage
- Bereitschaft zur Kontaktaufnahme nach Entlassung

Ausgeschlossen werden Patienten, die ausschließlich psychotherapeutisch behandelt werden und keine zusätzliche Begleitmedikation (nicht-psychiatrisch) einnehmen. Patienten, die von der geschützten Frauen-Station P12 auf die teilnehmenden, offenen Stationen verlegt werden, werden außerdem ausgeschlossen.

Bei Aufnahme ins Krankenhaus erfolgt eine ausführliche mündliche und schriftliche Aufklärung der Patienten über das geplante Projekt. Anschließend erhalten die Patienten schriftliches Informationsmaterial sowie das Formblatt zur Einverständniserklärung. Nach Ablauf einer Bedenkzeit von mindestens 24 Stunden wird das Formblatt wieder eingesammelt und der Patient entsprechend seiner Entscheidung in die Studie eingeschlossen oder nicht.

Um eine Überlappung von betreuten und nicht betreuten Patienten auf den Stationen zu verhindern, wird eine sequenzielle Kontrollgruppe verwendet: In den ersten 6 Monaten (KW 36/2012 – 09/2013) der Studie werden Patienten nur in die Kontrollgruppe aufgenommen, in den nächsten 6 Monaten der Studie (KW 14/2013 - KW 39/2013) werden die Patienten dann nur in die Interventionsgruppe

aufgenommen. Zwischen den Gruppenphasen erfolgt eine Wash-Out-Periode von 4 Wochen, um sicher zu stellen, dass die Patienten der Kontrollgruppe die stationäre Studienphase vor Beginn der Interventionsphase komplett beendet haben.

Ablauf der Betreuung:

1-2 Tage nach Aufnahme in die Klinik erfolgt bei allen Studienteilnehmern eine gründliche Medikamentenanamnese inklusive der Erstellung einer Medikationshistorie durch die Apothekerinnen. Jede Verordnung wir mit Hilfe des „Medication Appropriateness Index“ auf ABP geprüft [3]. Bei allen Patienten werden Nebenwirkungen und die Adhärenz mit Hilfe von validierten Fragebögen erfasst (siehe dazu 3. Studienbezogene Maßnahmen). Diese Fragebögen füllen die Patienten zusammen mit den Apothekerinnen 1-4 Tage nach Aufnahme, einen Tag vor und drei Monate nach Entlassung aus. Diese Ergebnisse werden zusammen mit dem Medikationsprofil auf AbP wie zum Beispiel fehlende Indikation und Dosierungsprobleme untersucht. Besonderes Augenmerk liegt hierbei auf der Ermittlung von Wechselwirkungen mit der Interaktionsdatenbank „Stockleys.“

In der Kontrollgruppe werden die AbP nur identifiziert und dokumentiert (Ausnahme aus ethischen Gründen: lebensbedrohliche oder massiv krankheitsverschlechternde Komplikationen).

In der Interventionsgruppe soll dagegen in Zusammenarbeit mit dem Arzt eine Lösung der AbP erarbeitet werden.

Die Adhärenz wird in beiden Gruppen während des Klinikaufenthaltes (1-4 Tage nach Aufnahme, sowie 1-2 Tage vor der Entlassung) und 3 Monate nach Entlassung erfasst. Patienten der Interventionsgruppe erhalten nach ca. 10 Tagen Klinikaufenthalt eine intensive Beratung über ihre Erkrankungen sowie eine ausführliche Aufklärung über die einzunehmenden Arzneimittel, deren Nutzen und Nebenwirkungen. Die Beratung beinhaltet ausführliche Informationen über:

- Psychiatrische Erkrankung des Patienten
- Begleiterkrankungen (z.B. Hypertonie, Diabetes mellitus)
- Welches Medikament für welche Erkrankung indiziert ist.
- Nebenwirkungen und besondere Wirkprofile, Wechselwirkungen
- Dosierungs- und Einnahmeregimes

Die Adhärenzsicherung bei Entlassung des Patienten von der Klinik in den ambulanten Bereich steht außerdem im Vordergrund (Schnittstellenmanagement). Da die Adhärenz vor allem nach der Entlassung gefährdet ist, erhalten Patienten der Interventionsgruppe einen Medikationsplan. Darüber hinaus wird diesen Patienten während der folgenden 3 Monate nach Entlassung aus der Klinik ein telefonisches Follow-up-Gespräch zur Adhärenzverbesserung durch eine Apothekerin angeboten. In diesem Telefongespräch soll nach Umstellung der Medikation durch den weiterbetreuenden Arzt, der Zufriedenheit mit der Therapie und der Adhärenz gefragt werden. Offene Fragen und individuelle Probleme der Patienten mit den Arzneimitteln können geklärt werden. Wenn nötig, wird eine ausführliche Beratung durchgeführt. Somit soll durch die medizinisch-pharmazeutische Betreuung die Schnittstelle zwischen stationärer und ambulanter Behandlung geschlossen werden

Studienbezogene Maßnahmen

Zur Erfassung der oben genannten Parameter füllen die Patienten der Interventions- und Kontrollgruppe folgende validierte Fragebögen in Zusammenarbeit mit der Apothekerin bei Aufnahme, ca. 1-2 Tage vor Entlassung und in der Nachbetreuungsphase ca. 3 Monaten nach Entlassung aus:

**Fragebogen Patient**		**Zu erfassende Parameter**
**UKU-SERS**	The Udvalg for Kliniske Undersøgelser Side Effect Rating Scale, nach Lingjaerde et al	Nebenwirkungen psychotroper Medikamente
**MARS**	Medication Adherence Report Scale-deutsche Version, nach Mahler et al	Medikamentenadhärenz
**DAI**	Drug Attitude Inventory, nach Hogan et al	Einstellung gegenüber Medikamenten, Adhärenz
**BphB (nur Interventionsgruppe)**	Beurteilung des pharmazeutischen Betreuungsservice	Zufriedenheit mit der pharmazeutischen Betreuung

Die Patientenzufriedenheit mit der pharmazeutischen Betreuung wird mit einem Fragebogen beurteilt, der für eine pharmazeutische Betreuungsstudie an Bypass-Patienten entwickelt worden ist. [9].

Als Leitfaden zur Identifikation Arzneimittel-bezogener Probleme, sowie zum Vergleich von Aufnahme-, Entlass- und Follow-up-Medikation in Hinblick auf Angemessenheit der Therapie wird der „Medication Appropriateness Index“ von den Apothekerinnen bewertet.

Primäre Endpunkte

- absolute Anzahl ungelöster Arzneimittelbezogener Probleme (AbP) pro Patient nach Abschluss des Studienplans des jeweiligen Patienten
- Angemessenheit der Therapie mithilfe des Medication Appropriateness Index (MAI)
- Adhärenz der Patienten

Sekundäre Endpunkte

- Anzahl gelöster und ungelöster AbP bei Entlassung und drei Monate danach zwischen Kontroll- und Interventionsgruppe
- Prävalenz von Nebenwirkungen (UKU Side Effect Rating Scale) bei Aufnahme, Entlassung und 3 Monate nach Entlassung
- Einfluss von Gewichtsänderungen/UAW allgemein auf die Einstellung (Entlassung, nach 3 Monaten)
- Einfluss von Gewichtsänderungen/UAW allgemein auf die Medikamentenadhärenz (Entlassung, nach 3 Monaten)
- Einfluss der Anzahl der Medikamente auf die Adhärenz zu Aufnahme, Entlassung und 3 Monate nach Entlassung

- Einfluss der Anzahl Medikament pro Patient auf die Anzahl der AbP bei Aufnahme, Entlassung und 3 Monate nach Entlassung
- Patientenzufriedenheit mit der pharmazeutischen Betreuung, nachdem die Patienten den Studienplan durchlaufen haben. (Fragebogen: Beurteilung des pharmazeutischen Betreuungsservices, erstellt von Dr. Sonja Koch im Rahmen ihrer Studie "Pilotstudie zur pharmazeutischen Betreuung von KHK-patienten, deskriptiv, nur Patienten der Interventionsgruppe)

Statistik

Mit Ausnahme der Patientenzufriedenheit mit der pharmazeutischen Betreuung (nur deskriptiv) werden für alle Messwerte (primäre und sekundäre Endpunkte) Mittelwert bzw. Median, Wertebereiche, Standardabweichung und Konfidenzintervall angegeben. Zum Nachweis statistisch bedeutsamer Unterschiede werden für kontinuierliche Messwerte bei vermuteten normalverteilten Werten der t-Test, bei vermuteten nicht-normalverteilte Werten der Mann-Whitney-U-Test durchgeführt. Bei binären Werten wird der Chi-Quadrat-Test durchgeführt. Die Mitarbeit eines Statistikers ist geplant.

Nutzen-Risiko-Abwägung

a. **Welcher Nutzen ist von den Ergebnissen der Studie zu erwarten**

aa) für die Versuchsteilnehmer?

Durch eine gründliche Anamnese sollen AbP wie z.B. Doppelverordnungen, Interaktionen und überflüssige Arzneimitteleinnahmen verhindert werden, um damit das Behandlungsregime für den Patienten so effektiv und einfach wie möglich zu gestalten.

Zudem wird der Therapieerfolg, also die Remission der Erkrankung, auch über einen längeren Zeitraum, ganz entscheidend von der Adhärenz des Patienten beeinflusst. Ziel dieses Projekts ist es, durch konsequente Schulung und Beratung zur ordnungsgemäßen Arzneimittelanwendung das Verständnis über Medikation und Erkrankung zu verbessern, um die Adhärenz zu erhöhen. Mehrfachgaben von Präparaten sollen, wenn möglich, durch Einmaldosen (Retardpräparate) ersetzt werden, um das Therapieschema der Patienten zu vereinfachen. Durch Aufklärung zu Vermeidungsstrategien für potentielle Nebenwirkungen und Behandlung von bzw. Umgang mit tatsächlich auftretenden Nebenwirkungen soll die Patientenzufriedenheit mit der Behandlung erhöht und damit ebenfalls ein wichtiger Beitrag zur Therapietreue geleistet werden.

ab) für die Heilkunde?

Doppelverordnung, Verschreibungen ohne Indikation oder auch unerwünschte Arzneimittelwirkungen durch Interaktionen können durch eine gezielte Anamnese verhindert und damit verbundene arzneimittelbezogene Kosten eingespart werden. Weiterhin führt ein langfristiger Therapieerfolg zu einer reduzierten Rezidivrate und damit zu einer reduzierten Rehospitalisierungsrate. Somit stellt die medizinisch-pharmazeutische Betreuung bei positiven Studienergebnissen einen Ansatz zur

Verbesserung der Versorgungsqualität sowie zum wirtschaftlichen Ressourceneinsatz dar.

ac) für die Wissenschaft (z.B. Ergebnisse, die nicht unmittelbar therapeutischen Zwecken dienen)?

Erstmalig versucht ein Apotheker in Deutschland auf einer psychiatrischen Station durch medizinisch-pharmazeutische Betreuung AbP zu reduzieren und die Patientenadhärenz zu verbessern, um somit den Therapieerfolg zu unterstützen. Durch das Design der Studie mit Kontrollgruppe kann erstmals die Reduktion der Arzneimittel-bezogenen Probleme im Vergleich zur Standardbetreuung aufgezeigt werden. Zusätzlich soll der Einfluss einer pharmazeutischen Nachbetreuung der Patienten auf die Adhärenz nach Entlassung aus der Klinik mit Daten belegt werden.

b. **Mit welchem Risiko ist die Studie für die Versuchsteilnehmer verbunden? Welcher Art sind die Risiken?**

Die Studie ist mit keinem Risiko verbunden.

Literatur

1. Foppe van Mil, J.W., et al., *Drug-related problems in public pharmacies.* Arzneimittelbezogene probleme in der öffentlichen apotheke, 2001. **146**(16): p. 24-30.
2. World-Health-Organization, *Adherence to long-term therapies: evidence for action.* . Geneva, 2003.
3. Hanlon, J.T., et al., *A method for assessing drug therapy appropriateness.* Journal of Clinical Epidemiology, 1992. **45**(10): p. 1045-1051.
4. Jacobi, F., M. Klose, and H.U. Wittchen, *[Mental disorders in the community: healthcare utilization and disability days].* Bundesgesundheitsblatt Gesundheitsforschung Gesundheitsschutz, 2004. **47**(8): p. 736-44.
5. Bühren, A., et al., *Mental disorders: All specialties are needed.* Psychische erkrankungen - Alle fachgebiete sind gefordert, 2008. **105**(17): p. A880-A884.
6. Kampfhammer, H., *Psychische Störungen bei somatischen Krankheiten.* Psychiatrie, Psychosomatik und Psychotherapie, Springer-Verlag, 2011. **4. Auflage.**
7. Valenstein, M., et al., *Pharmacy data identify poorly adherent patients with schizophrenia at increased risk for admission.* Med Care, 2002. **40**(8): p. 630-9.
8. Melfi, C.A., et al., *The effects of adherence to antidepressant treatment guidelines on relapse and recurrence of depression.* Archives of General Psychiatry, 1998. **55**(12): p. 1128-1132.
9. Klopotowska, J.E., et al., *On-ward participation of a hospital pharmacist in a Dutch intensive care unit reduces prescribing errors and related patient harm: an intervention study.* Crit Care, 2010. **14**(5): p. R174.
10. Koch, S., *Pharmazeutische Betreuung von Patienten mit koronarer Herzkrankheit nach aortokoronarer Bypassoperation - eine Pilotsudie -.* Dissertation, 2009.
11. Dolder, C., et al., *Pharmacist interventions in an inpatient geriatric psychiatry unit.* American Journal of Health-System Pharmacy, 2008. **65**(19): p. 1795-1796.
12. Canales, P.L., P.G. Dorson, and M.L. Crismon, *Outcomes assessment of clinical pharmacy services in a psychiatric inpatient setting.* American Journal of Health-System Pharmacy, 2001. **58**(14): p. 1309-1316.
13. Finley, P.R., et al., *Impact of a collaborative care model on depression in a primary care setting: A randomized controlled trial.* Pharmacotherapy, 2003. **23**(9 I): p. 1175-1185.
