# Supplementary material for: Pharmacist-Led Medication Reviews to Identify and Collaboratively Resolve Drug-Related Problems in Psychiatry – A Controlled, Clinical Trial
Source: PLoS One. 2015 Nov 6;10(11):e0142011. doi: 10.1371/journal.pone.0142011 (PMC4636233; doi:10.1371/journal.pone.0142011)
Supplement: S2 Protocol — (PDF) [file pone.0142011.s004.pdf]

Pharmaceutical Care of psychiatric inpatients - Study protocol

Background

In Germany, the 12-month-prevalence of psychiatric disorders is 37 % for women and 25,3 % for men. In recent years this number has increased, especially with depressive disorders. It is extremely important to get an exact and complete anamnesis of the medication of these patients, because psychiatric disorders are often accompanied by comorbidity and polypharmacy- The risk of DRP rises exponentially. Furthermore, the rate of therapy discontinuation is very high due to the special mode of action of the drugs (onset of effect after 2 -3 weeks, but occurrence of side effects immediately). The number of adherent patients varies from 20 to 70 % even after the introduction of modern preparations. Adherence is a very important component of an adequate and successful therapy of psychiatric disorders. The risk of repeated hospitalization of a non-adherent schizophrenic patient is according to Valenstein et al 2.4 times higher compared to an adherent schizophrenic patient. The same applies to patients with affective disorders. Discontinuation of therapy of patients with affective disorder leads to an increase of 77 % in the risk of relapse. The concept of the pharmaceutical care is the basis for reduction of DRP as well as for the improvement of adherence. Pharmaceutical care is based on the intensive cooperation of the patient, the doctor in charge and the pharmacist. The aim is to instruct the patient together with the doctor in order to apply the medication securely and appropriately, to recognize and to solve possible DRP, to optimize the drug therapy and resulting from this, to improve the patient's quality of life.

A Dutch working group with Joanna Klopotowska examined the impact of a ward pharmacist on the number of DRP in an intensive care unit. A reduction of DRP from 190.5 per 1000 observed patient days down to 62.5 per 1000 days could be established. A study on pharmaceutical care of patients with coronary heart disease proved a significant increase in adherence after an aortocoronary bypass surgery. After 12 months, adherence in the intervention group was 90 % (base value 88 %) and in the control group 71 % (base value 81 %). The care concept included apart from talks with a hospital pharmacist during the hospital stay also follow-up talks during the outpatient phase as well as the handing out of written information.

The present study on pharmaceutical care of psychiatric patients in hospital is to investigate if similarly positive effects on the number of DRP as well as on the adherence of medication in this group of patients can be achieved. Validated questionnaires are used for quantification.

To our knowledge no comparable studies with psychiatric patients exist. A first project in Great Britain covered 282 interventions by pharmacists in ward. In a study on care in the USA, 62 % of the patients experienced a more than 30 % improvement in the Brief Psychiatric Rating Scale. In another study, an improvement of 19% in adherence was achieved. With the present, planned study, for the first time, pharmaceutical care by pharmacists in ward in close collaboration with a clinician is to be implemented in a German psychiatric hospital. Additionally, the benefit of the given care of a pharmacist has to be quantitatively evaluable.

Aim of the project

The project aims to assess by a clinical study if pharmaceutical care implemented by the integration of a pharmacist on ward in the multidisciplinary team of the psychiatric university hospital can

reduce and enhance the following parameters respectively: the number of drug-related problems (DRP), the appropriateness of therapy and the adherence, in particular after discharge.

Methods

The planned study on pharmaceutical care is a prospective open interventional study with sequential control and intervention groups. Psychiatric patients from the wards P21 and P31 of the Psychiatric and Psychotherapeutic department of the University Hospital Erlangen are included regardless of the diagnosis.

Further inclusion criteria must be met

- Age ≥ 18
- Capability to fill in questionnaires independently
- Capability to understand spoken and written German
- Capability to give consent
- Inpatient stay in the Psychiatric Clinic Erlangen ≥7 days
- Willingness to get into contact after discharge

Patients are excluded who are being treated only psychotherapeutical und do not take any additional co-medication (non-psychiatric). Patients, who are transferred from the acute women's ward to the participating open wards, are also excluded.

When admitted to hospital, the patient receives detailed written information about the planned project and a form for the declaration of consent. After a period of reflexion of at least 24 hours, the form is collected and the patient is included in the study or not according to his decision.

To avoid an overlap of pharmaceutical cared and patients without pharmaceutical care on the wards, a sequential control group is used: During the first six months (cw 26/12 – cw 09/13) of the study, patients are only admitted to the control group, in the following 6 months (cw 14/2013 – cw 39/2013) only to the intervention group. Between the two phases, a wash-out period of 4 weeks takes place in order to guarantee that the patients of the control group are discharged before the start of the intervention phase

Pharmaceutical care plan

1 – 2 days after admission to hospital, a thorough medication review including a medical history of all study participants is taken by the pharmacists. Every prescription is examined by means of the "Medication Appropriateness Index. Side effects and adherence of all patients are determined on the basis of questionnaires.

The patients fill in these questionnaires together with the pharmacists one to four days after admission, one day before and three months after discharge. Together with the medication profile the results of these questionnaires are examined on DRP such as missing indication, side-effects or non-adherence. Particular attention is paid to the determination of interactions with the interaction

database “Stockley’s”. In the control group, the DRP are only identified and documented (exception for ethical reasons: life-threatening or disease worsening complications).

In the intervention group, a solution of the DRP is to be worked out together with a physician.

The adherence in both groups is determined during the hospital stay (1-4 days after admission, and 1-2 days before discharge) and 3 months after discharge. Ten days after admission, the patients of the intervention group receive an intensive consultation concerning their illness as well as detailed information on their medication, as well as benefit and side effects of the drugs. The consultation consists of detailed information on

- Psychiatric disease of the patient
- Accompanying diseases (hypertension, diabetes mellitus)
- Which medication for which disease is indicated
- Dosage- and taking regime

The securing of adherence when patients are discharged from hospital into the outpatient sector is paramount (seamless care). The adherence especially being jeopardized after discharge, patients of the intervention group receive a discharge medication plan. Furthermore, these patients are offered a follow-up telephone call by the pharmacist in order to improve adherence for the following 3 months. During this call, questions are to be asked on change of medication by their physician, the satisfaction with the treatment and the adherence. Open questions and individual problems of the patients with medications can be clarified. If necessary, a thorough consultation is given. Thus, the interface between hospitalized and outpatient treatment is to be closed by pharmaceutical care.

Outcome assessment

In order to determine a.m. parameters, the patients of the intervention- and control group fill in the following validated questionnaires together with the pharmacist on the day of admission, 1-2 days before discharge, and 3 months after discharge during the follow-up phase.

**Questionnaire**		**Parameter**
**UKU**	The Udvalg for Kliniske Undersøgelser Side Effect Rating Scale, nach Lingjaerde et al	Side-effects of psychotropic drugs
**MARS**	Medication Adherence Report Scale-German version (Mahler et al)	Adherence
**DAI**	Drug Attitude Inventory (Hogan et al)	Adherence and attitude
**Beurteilung des pharmazeutischen Betreuungsservices (BphB, intervention group)**	Evaluation of the pharmaceutical care service	Satisfaction with pharmaceutical care

The patient satisfaction concerning the pharmaceutical care is judged by a questionnaire developed for a study of pharmaceutical care with bypass patients [9].

The pharmacists use the “Medication Appropriateness Index” as a guideline in order to identify DRP and to compare admission-, discharge and follow-up medication in respect to appropriateness of the therapy.

Primary Outcomes

- Number of unsolved Drug related problems (DRP) per patient after the patient finished the study plan
- Appropriateness of therapy measured with the Medication Appropriateness Index
- Patient adherence

Secondary Outcomes

- Number of solved and unsolved DRP at discharge and 3 months after discharge between control- and intervention group
- Prevalence of side effects assessed with the UKU side effect rating scale at admission, discharge and 3 months after discharge
- Influence of assessed weight changes/ADE on the attitude according to psychotropic drugs (discharge, 3 months after discharge)
- Influence of assessed weight changes/ADE on adherence of the patients (discharge, 3 months after discharge)
- Influence of the number of medication on adherence (admission, discharge, 3 months after discharge)
- Influence of the number of medication on the number of detected DRP (admission, discharge, 3 months after discharge)
- Patients' satisfaction with pharmaceutical care after the patients terminate the study plan (Questionnaire: "Assessment of the pharmaceutical care service", developed by PhD Sonja Koch in the course of her study "A pilot study regarding pharmaceutical care of patient with coronary heart disease", descriptive, only patients of the intervention group)

Statistics

With the exception of the patient’s satisfaction with the pharmaceutical care (only descriptive) average values resp. median, value ranges, standard deviation and confidence interval will be specified for all measured values (primary and secondary end points). In order to determine statistically important differences, for continuous measured values, T-test will be performed when values are presumably normally distributed, the Mann-Whitney-U-Test, when presumably values are not normally distributed. In the case of binary values the Chi-Quadrat-Test will be performed. The use of the assistance of a statistician is intended.

Risk-benefit relation

a) Which benefit is to be expected of the results of the study?

aa) A thorough medication review including a complete medication history can prevents DRP such as double prescription, interactions and unnecessary drug intake in order to make the treatment regime of the patient as effective and simple as possible. Moreover, the therapy success, in detail the remission of the disorder, is significantly influenced by the patient's adherence over a long period. The aim of the project is to improve the understanding of medication and disease by consequently training and informing the patients about proper pharmaceutical drug usage in order to increase adherence. If possible, sustained release drugs will replace multiple doses, in order to simplify the patient's therapy scheme. The patient's satisfaction with the treatment is to be improved by informing on avoidance strategies concerning possible side effects and on treatment respectively handling of actually occurring side effects.

Ab) for the medicine

Double prescriptions, prescriptions without indication as well as side effects of drugs through interactions can be prevented by a targeted medication review likely leading to the saving of medicine-related costs. Furthermore a long-termed therapy success leads to a reduced recurrence rate and hence to a reduction in the rate of re-hospitalization. Thus, the pharmaceutical care represents in case of positive results an approach to improve the quality of care as well as to use resources more efficiently.

Ac) for the science (e.g. results which do not serve directly therapeutical purposes)

It is for the first time that in Germany a pharmacist in a psychiatric ward tries to reduce DRP by pharmaceutical care and to improve the patient's adherence in order to support therapy success. Due to the design of the study with a control group, a reduction of DRP compared to standard care can be shown for the first time.

b) What are the risks of the study for the trial participants? What kind of risks?

The study does not involve any risks.

Literature

1. Foppe van Mil, J.W., et al., *Drug-related problems in public pharmacies.* Arzneimittelbezogene probleme in der öffentlichen apotheke, 2001. **146**(16): p. 24-30.
2. World-Health-Organization, *Adherence to long-term therapies: evidence for action.* . Geneva, 2003.
3. Hanlon, J.T., et al., *A method for assessing drug therapy appropriateness.* Journal of Clinical Epidemiology, 1992. **45**(10): p. 1045-1051.
4. Jacobi, F., M. Klose, and H.U. Wittchen, *[Mental disorders in the community: healthcare utilization and disability days].* Bundesgesundheitsblatt Gesundheitsforschung Gesundheitsschutz, 2004. **47**(8): p. 736-44.
5. Bühren, A., et al., *Mental disorders: All specialties are needed.* Psychische erkrankungen - Alle fachgebiete sind gefordert, 2008. **105**(17): p. A880-A884.
6. Kampfhammer, H., *Psychische Störungen bei somatischen Krankheiten.* Psychiatrie, Psychosomatik und Psychotherapie, Springer-Verlag, 2011. **4. Auflage.**
7. Valenstein, M., et al., *Pharmacy data identify poorly adherent patients with schizophrenia at increased risk for admission.* Med Care, 2002. **40**(8): p. 630-9.
8. Melfi, C.A., et al., *The effects of adherence to antidepressant treatment guidelines on relapse and recurrence of depression.* Archives of General Psychiatry, 1998. **55**(12): p. 1128-1132.
9. Klopotowska, J.E., et al., *On-ward participation of a hospital pharmacist in a Dutch intensive care unit reduces prescribing errors and related patient harm: an intervention study.* Crit Care, 2010. **14**(5): p. R174.
10. Koch, S., *Pharmazeutische Betreuung von Patienten mit koronarer Herzkrankheit nach aortokoronarer Bypassoperation - eine Pilotsudie -.* Dissertation, 2009.
11. Dolder, C., et al., *Pharmacist interventions in an inpatient geriatric psychiatry unit.* American Journal of Health-System Pharmacy, 2008. **65**(19): p. 1795-1796.
12. Canales, P.L., P.G. Dorson, and M.L. Crismon, *Outcomes assessment of clinical pharmacy services in a psychiatric inpatient setting.* American Journal of Health-System Pharmacy, 2001. **58**(14): p. 1309-1316.
13. Finley, P.R., et al., *Impact of a collaborative care model on depression in a primary care setting: A randomized controlled trial.* Pharmacotherapy, 2003. **23**(9 I): p. 1175-1185.
